# Tumor-dependent myeloid and lymphoid cell recruitment in genO-BRGSF-HIS mice: a novel tool for evaluating immunotherapies

**DOI:** 10.3389/fimmu.2025.1624724

**Published:** 2025-09-17

**Authors:** Gaëlle H. Martin, Siham Hedir, Florent Creusat, Alexis Gonon, Amélie Marguier, Perrine Martin-Jeantet, Lise Nouveau, Laura Cons, Florence Renart-Depontieu, Valery Moine, Marc Derive, Yacine Cherifi, Margarida T. Grilo Ruivo, Fabiane Sônego, Kader Thiam

**Affiliations:** ^1^ genOway, Lyon, France; ^2^ Light Chain Bioscience - Novimmune SA, Geneva, Switzerland; ^3^ Inotrem SA, Paris, France

**Keywords:** humanized preclinical model, tumor microenvironment, human myeloid cells, human lymphoid cells, genO-BRGSF-HIS, immunotherapy

## Abstract

**Objectives:**

Preclinical models that accurately recapitulate the human immune response, particularly within the tumor microenvironment (TME), are needed for the translational and predictive testing of new therapies. Here, we examine whether the genO-BRGSF-HIS model—characterized by robust reconstitution of both human lymphoid and myeloid cells following engraftment with CD34^+^ cord blood cells—could be a translatable mouse model for human tumor biology and a relevant platform for evaluating novel immunotherapies.

**Methods:**

genO-BRGSF mice were reconstituted with human CD34^+^ cord blood cells (genO-BRGSF-HIS) and treated with exogenous human Flt3 ligand (hFlt3L). Myeloid and dendritic cell functionality was analyzed following treatment with different compounds (TLR agonists, TREM1 agonist, STING agonist, or T-cell engagers) and following the implantation of different tumor cell lines (MDA-MB-231, A549, HPAF-II).

**Results:**

We show that myeloid, dendritic and lymphoid cells (including NK and γδ T cells) are functional and recruited into the TME in genO-BRGSF-HIS mice implanted with different tumor cell lines, and that different immune cell populations are activated and get polarized within the TME. The composition of the TME is dependent on tumor type and tumor burden, demonstrating plasticity in the crosstalk between the human immune system and the tumor cells. Furthermore, we observed polarization of the cells recruited to the TME, as well as a wide diversity of recruited cell populations, suggesting that this model reproduces human physiopathology in the context of cancer. Based on the recruitment of the different cell populations according to tumor type, we also demonstrate that this model can be used for testing new therapies targeting lymphoid cells, such as T-cell engagers.

**Conclusions:**

genO-BRGSF-HIS mice do not exhibit adverse effects associated with the development of human lymphoid and myeloid cells following CD34^+^ cord blood cell reconstitution, and their extended lifespan allows for longer experimental study windows. Overall, we show that this model develops functional myeloid and lymphoid cells which are recruited to the TME, making it a valuable tool for testing new immunotherapies that modulate the interaction between the tumor and the immune system.

## 1 Introduction

The role of immune cells in tumor initiation and maintenance has received considerable attention in recent decades, particularly the role of the TME in cancer progression. Lymphoid, myeloid and dendritic cells (DCs) are known to be involved in tumor establishment and progression, and to influence therapeutic outcomes. In particular, T cells have been described to play a dual role in tumor immunity: cytotoxic T cells are crucial for eliminating tumor cells, while regulatory T cells (Tregs) foster an immunosuppressive environment that supports tumor growth. Consequently, much effort has been devoted to harnessing the T cell’s unique ability to selectively target and kill tumor cells for the development of novel anti-tumor therapies. However, these approaches still face many challenges, such as an immunosuppressive environment within the TME and T-cell exhaustion, which limit the efficacy and safety of these therapies.

The myeloid cell population is also known to play an essential role in modulating the tumor response, and the balance between pro- and anti-tumor function of myeloid cells is a critical factor in determining the overall response to treatment. The infiltration of certain subsets of myeloid cells can contribute to the establishment of an immunosuppressive environment in the tumor, promoting immune evasion and supporting tumor growth. Due to these characteristics, several strategies focus on targeting the myeloid cell population for cancer treatment. Modern immunotherapies are designed not only to target cancer cells directly but also to modulate the immune system in highly specific ways. A key strategy involves co-targeting both lymphoid and myeloid cells to enhance anti-tumor immunity while overcoming the immune evasion mechanisms employed by tumors (1–4). Other strategies involve the reprogramming of immune cells within the tumor microenvironment, shifting their function from an immunosuppressive to a pro-inflammatory, anti-tumor state, or the selective depletion of Tregs, as these cells contribute to immune suppression and tumor progression. The development of conditionally active monoclonal antibodies has also shown promising results, as these antibodies remain inactive in healthy tissues but become selectively activated within the tumor microenvironment (2, 3, 5). This last strategy helps to improve therapeutic efficacy while minimizing off-target toxicity, addressing one of the major challenges of systemic immunotherapies. These advances mark a shift toward the use of more precise immunotherapeutic strategies, leveraging the complexity of the immune system to achieve better safety and efficacy profiles in cancer treatment.

To facilitate the development and improvement of these therapies, significant efforts have been made to develop translational mouse models. An increased number of mouse models with human immune systems (HIS) are being developed for the study of human diseases (6, 7). Humanized mouse models can be generated by engrafting immunodeficient mice with CD34^+^ hematopoietic stem cells (HSCs), human fetal liver and thymus tissues, or human peripheral blood mononuclear cells (PBMCs). However, depending on the approach used, these mice have different characteristics, and their human immune system develops to different levels (8). For example, PBMC-reconstituted mice are known to develop graft-versus-host disease (GvHD) due to a predominance of T cells, often exhibiting an activated or memory phenotype. In such models, myeloid cells are underrepresented (9), which constitutes another major limitation to investigate immunotherapies. To address this shortcoming, new models with enhanced human cytokine expression have been developed, resulting in the presence of functional myeloid cells upon humanization (10–12). However, despite the presence of functional DCs and monocytes, these models still have limitations. For example, the NSG-SGM3 and NOG-EXL models develop severe clinical signs such as macrophage activation syndrome, liver lesions, or hemophagocytic lymphohistiocytosis (HLH), resulting in a reduced lifespan (12–14). MISTRG mice were also shown to develop anemia, resulting in a short experimental window of 2–3 weeks (10). One recent model expressing human Flt3L has also been described to develop human myeloid and dendritic cells upon reconstitution with human CD34^+^ cells (15). Despite showing improved numbers of DCs, the numbers of the different DC populations sharply fall at around 16–18 weeks, potentially due to the supraphysiological expression of human hFlt3L, leading to an exhaustion of the human hematopoietic stem cells.

The BRGS model (BALB/c Rag2^tm1Fwa^ IL2Rγ_c_^tm1Cgn^ SIRPα^NOD^) is a strain of immunocompromised mice developed on a BALB/c background. These mice have a high resistance to radiation, a functional complement system, and are highly permissive to engraftment with human hematopoietic stem cells (16). This model has since been established as a valuable tool for testing the efficacy of anti-cancer therapeutics in patient-derived xenografts (PDX) and cell line-derived xenografts (CDX), such as those targeting PD-1 (17–20). However, these mice often have a suboptimal production of human myeloid cells. This led to the development of the BRGSF model, here called genO-BRGSF, which has a deficient murine myeloid cell compartment due to targeted inactivation of the fms related receptor tyrosine kinase 3 (*Flt3*) gene. This model was shown to develop lymphoid, myeloid and dendritic cell compartments upon treatment with exogenous human Flt3 ligand (hFlt3L) (21), and to maintain long-term engraftment without developing GvHD (22). Here we show that the myeloid cells in this model are functional, and are recruited to the TME of different tumors alongside lymphoid cells such as natural killer (NK) cells, where they become polarized and express different activation markers. Interestingly, the TME composition of different tumors varies according to tumor type and tumor burden, recapitulating what is observed in patients (23). By enabling the study of the complex tumor-immune cells interactions *in vivo*, we demonstrate that the genO-BRGSF-HIS mouse could be a valuable tool for understanding tumor-immune dynamics and for preclinical evaluation of novel therapeutic strategies.

## 2 Results

### 2.1 genO-BRGSF-HIS mice display a stable and long-term engraftment with human CD34^+^ cells and develop a myeloid compartment that can be re-stimulated

**Figure 1:**
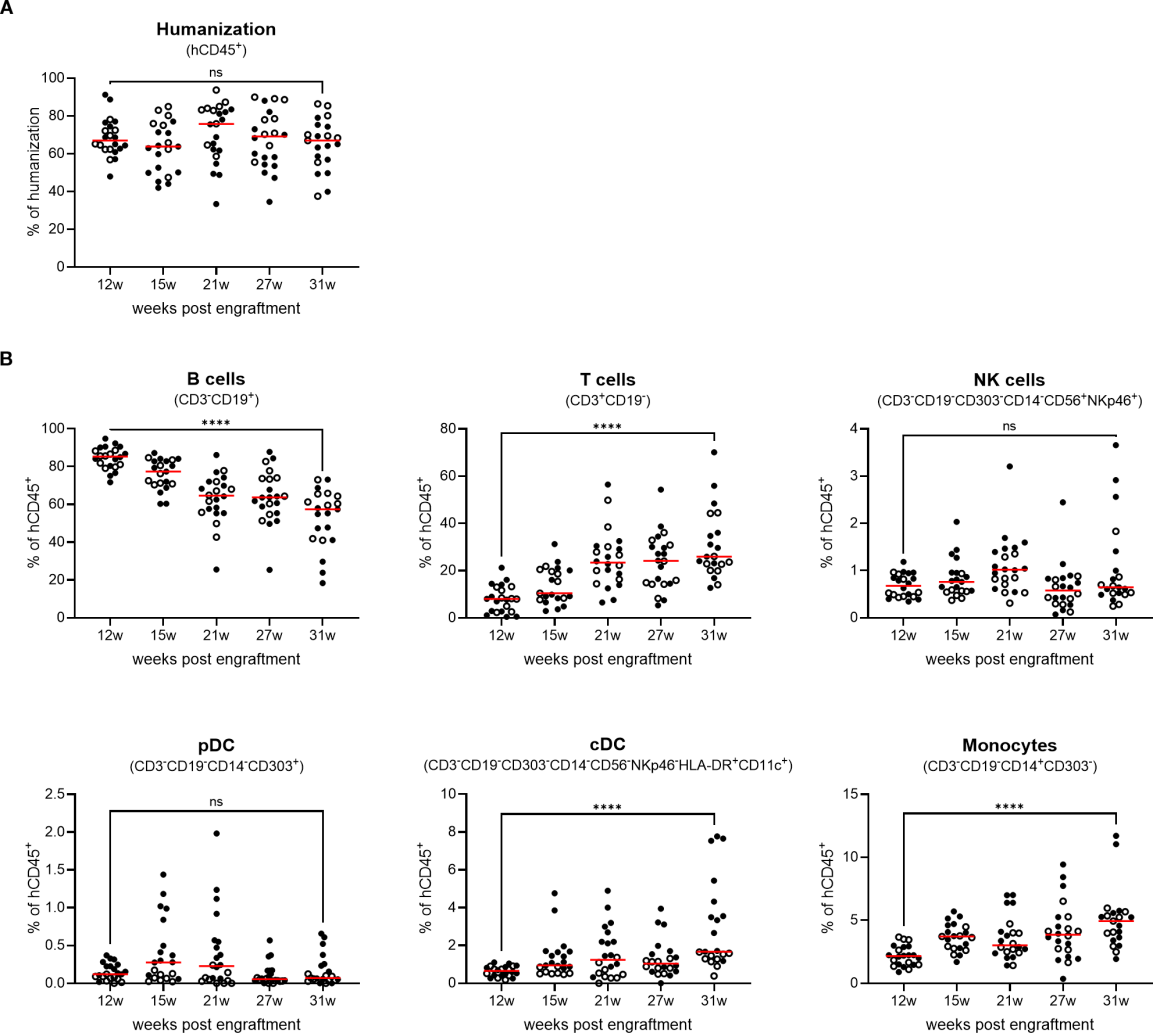
genO-BRGSF-HIS mice develop stable lymphoid, myeloid, and dendritic compartments for 31 weeks, and the myeloid and dendritic compartments can be restimulated. **(A)** The rate of humanization was measured in the blood over 31 weeks (w) in genO-BRGSF-HIS mice (n=22) engrafted with hCD34^+^ cells from two donors (black circles and white circles). Percentage of hCD45^+^ cells relative to total live leukocytes is shown; horizontal lines represent the mean. **(B)** Quantification of the different cell populations following hCD34^+^ HSC engraftment over 31 weeks, represented as a percentage relative to human CD45^+^ cells; horizontal lines represent the mean. Data in **(A, B)** were analyzed using a Mann Whitney test. ****P<0.0001.

**Figure 2:**
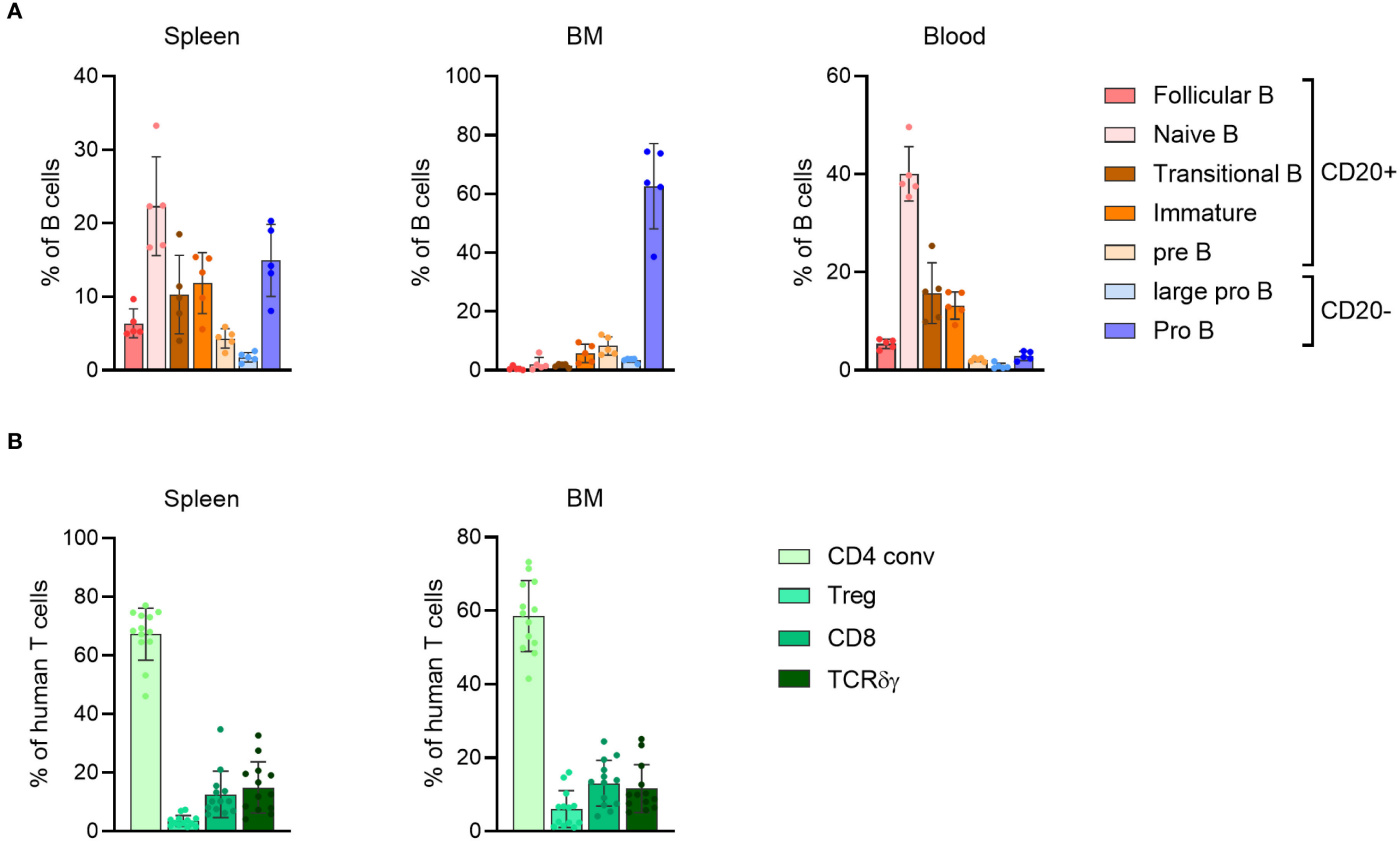
genO-BRGSF-HIS mice develop different subsets of lymphoid cells. **(A)** Quantification of the B cell subpopulations in the spleen, bone marrow (BM), and blood of hFlt3L-treated genO-BRGSF-HIS mice at 19 weeks of age (n=5, 3 hCD34^+^ cell donors). Quantifications are represented in percentages relative to hCD45^+^ cells. **(B)** Quantification of the T cell subpopulations in the spleen or bone marrow (BM) of genO-BRGSF-HIS mice at 18 weeks of age (n=13, 6 hCD34^+^ cell donors), represented in percentages relative to hCD45^+^ cells. Error bars represent standard deviation individual points represent individual mice.

The presence of human T cells in the BRGSF-A2-HIS model containing a human leukocyte antigen serotype A2 (HLA-A2) class I transgene has been shown to be maintained for up to 55 weeks post engraftment in the blood and lymphoid tissues (22). This assessment, however, was performed across different cohorts of mice, and only analyzed the maintenance of T cells throughout time. To gain an insight into the persistence of the immune system engraftment in mice, a longitudinal analysis of different cell populations was performed in genO-BRGSF mice reconstituted with human CD34^+^ cord blood cells (genO-BRGSF-HIS). The humanization rate was analyzed over the course of 31 weeks post engraftment, which corresponds to the timeframe often used for experimental testing with these mice. In agreement with what had previously been observed (22), the levels of humanization in the blood stayed constant throughout time, with the average humanization rate varying between 60% and 75% (**Figure 1A**). This suggests that human progenitor cells are still present throughout this period, enabling the maintenance of a stable engraftment. Despite no significant changes in the percentage of hCD45^+^ cells, a decrease in the absolute numbers was observed over time, which could be explained by the decreasing numbers of B cells (**Supplementary Figures 1A, B**). In line with previous observations (21, 24, 25), T, B, and NK cells, as well as dendritic cells and monocytes, developed in these mice. In this longitudinal study, B cells were the most predominant population of immune cells, followed by T cells, as previously described in genO-BRGSF-HIS mice and other hCD34^+^ HSC-reconstituted models (**Figure 1B**; **Supplementary Figure 1B**). A change in the ratio of T to B cells was observed over time (~1.5-fold decrease in the percentage of B cells and ~3-fold increase in T cells), as well as an increase in the percentages of cDCs and monocytes of ~5-fold and ~2-fold, respectively (**Figure 1B**; **Supplementary Figure 1B**). Further characterization of the subpopulations of B and T cells showed that their relative proportions varied across the analyzed organs (**Figures 2A, B**; **Supplementary Figures 2A–C**). As expected, the bone marrow was mainly composed of pro-B cells, while the spleen and blood contained a higher percentage of mature B cells (**Figure 2A**; **Supplementary Figure 2B**). T-cell distribution was similar between the two organs analyzed (**Figure 2B**; **Supplementary Figure 2C**). In accordance with what has been previously published, development of γδ T cells was also observed (25), and comprised around 15% and 12% of the T-cell population in the spleen and bone marrow, respectively (**Figure 2B**; **Supplementary Figure 2C**).

**Figure 3:**
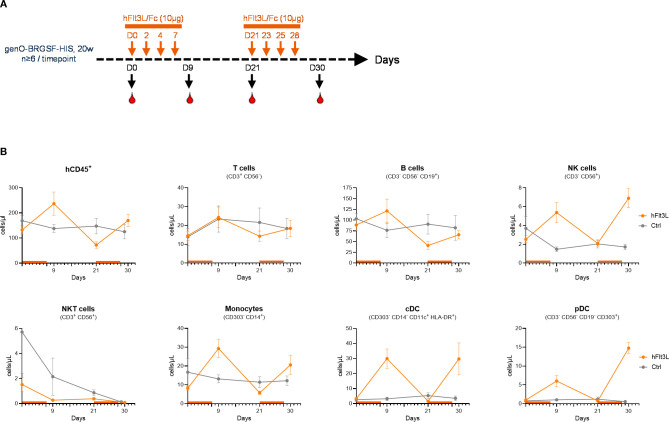
The myeloid and dendritic compartments in genO-BRGSF-HIS mice can be restimulated. **(A)** Scheme illustrating the experimental outline for the two hFlt3L boosts. **(B)** Quantification of different cell subpopulations in the blood over 30 days, following one and two hFlt3L or vehicle (ctrl) treatments. At least 6 mice per timepoint were analyzed, engrafted with hCD34^+^ cells from 3 donors. Error bars represent standard error of the mean.

Characterization of the myeloid and dendritic cell population demonstrated that exogenous treatment with hFlt3L resulted in a significant increase in the frequency and numbers of dendritic cells, including pDCs and cDCs (cDC1 and cDC2) (**Supplementary Figures 3A, B**), corroborating previous reports (11, 21, 24). To further investigate the impact of hFlt3L treatment, we monitored the dynamics of myeloid and dendritic cell proliferation in the blood and observed that this increase was transient in naïve mice, lasting up to three days (data not shown). Indeed, the number of monocytes, cDCs and pDCs increased two days after the final injection of hFlt3L and remained at basal levels following their decrease (**Figures 3A, B**). Given this finding, we tested whether the myeloid and dendritic compartments could be re-stimulated and observed that they remained responsive to hFlt3L stimulation, as a second round of treatment led to a similar increase in the number of myeloid and dendritic cells (**Figure 3B**). This effect was particularly evident in dendritic cells, as evidenced by a 19-fold and 21-fold increase in the numbers of cDCs and pDCs respectively, following the second hFlt3L treatment. Although in small percentages (0.5–1%), NK cells were also detected in the blood of naïve genO-BRGSF-HIS mice (**Figure 1A**) and increased following hFlt3L stimulation (**Figure 3B**) as previously reported (21).

Altogether, this data shows that hCD34^+^ cell engraftment in genO-BRGSF mice results in a stable development of lymphoid and myeloid cells which lasts for at least 31 weeks with no observed side effects or development of GvHD, suggesting that there is no exhaustion of the human hematopoietic stem cells. hFlt3L treatment transiently boosts the human myeloid and dendritic cell compartments and we show that these compartments can be re-boosted.

### 2.2 genO-BRGSF-HIS’ human myeloid cells are functional and feature responsive pattern-recognition receptors

**Figure 4:**
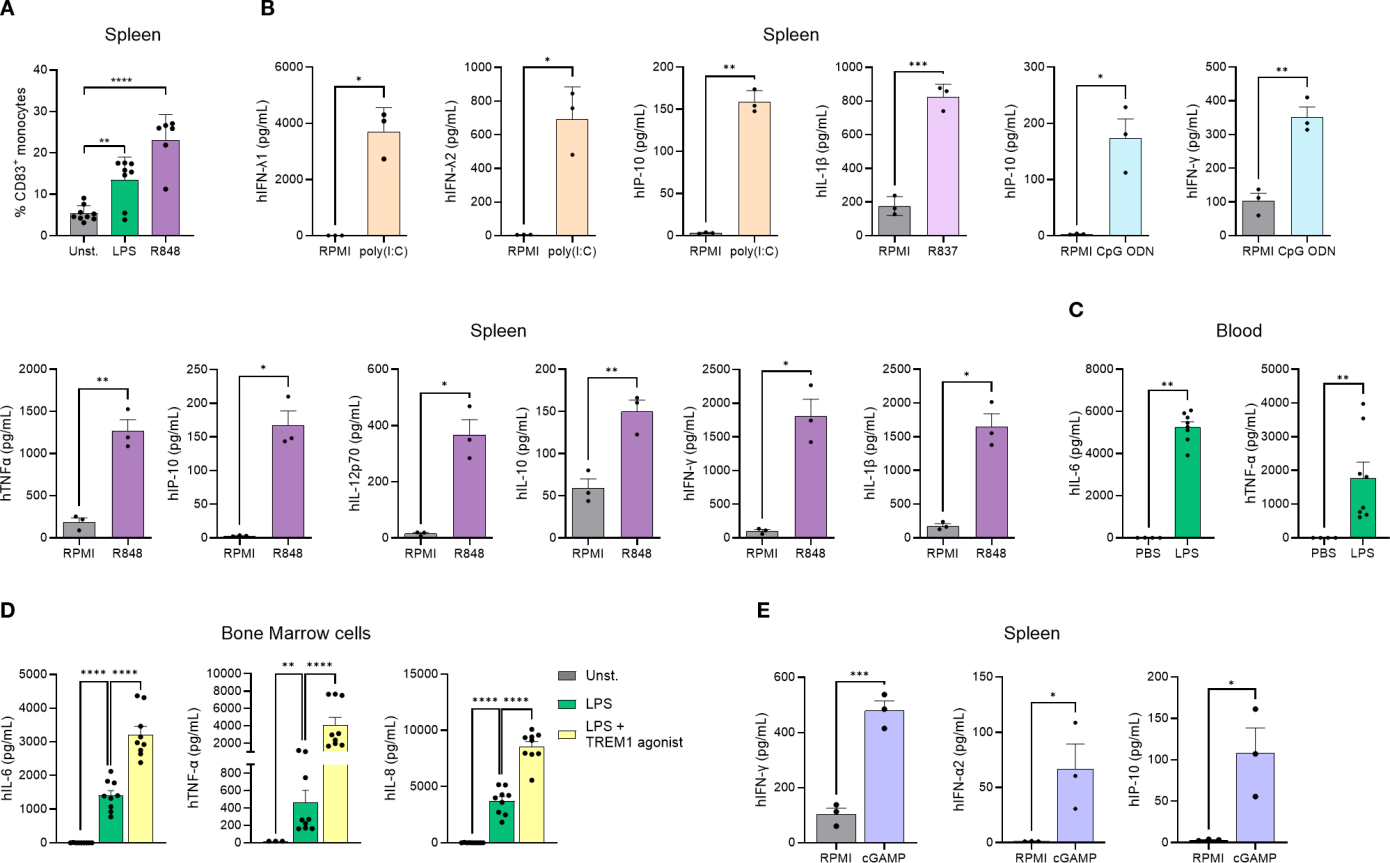
Human immune cells in the genO-BRGSF-HIS model express different PRRs and are functional. **(A)** Activation of monocyte-derived dendritic cells following TLR4 or TLR7/8 stimulation. Monocytes (hCD14^+^) isolated from 8 hFlt3L-treated genO-BRGSF-HIS mice were treated with LPS at 0.1 µg/mL or R848 (Resiquimod) at 250 µg/mL, or left unstimulated (Unst). CD83 expression was measured 24 hours later by cytometry. Data were analyzed with a one-way ANOVA. **(B)** *Ex vivo* human splenocytes were treated with 10 µM of CpG ODN; 10 µg/mL of Poly(I:C), 10 µg/mL of R837, or 10 µg/mL of R848, and cytokine production was measured by LEGENDplex. **(C)** Blood samples were collected after treating hFlt3L-treated genO-BRGSF-HIS mice (n=4) with 2 mg/kg of LPS, and cytokine release was measured by LEGENDplex. **(D)** *Ex vivo* bone marrow cells from 4 hFlt3L-treated mice were treated with 0.1 μg/mL LPS or 0.1 μg/mL LPS and a TREM1 agonistic antibody (10 μg/mL). Cytokine secretion was measured six hours later by LEGENDplex. **(E)** *Ex vivo* splenocyte cultures from 4 hFlt3L-treated genO-BRGSF-HIS mice treated with 10 µg/mL of 2'3' cGAMP. Cytokine release was measured by LEGENDplex. In graphs **(A, C, D)**, single dots represent replicates, and error bars represent standard error of the mean. In graphs **(B, E)**, 3 HSC donors were used, and 3 mice were used per donor (9 mice in total). Individual points represent pooled mice per donor. Bars represent standard deviation. Data in graphs **(B, E)** were analyzed with an unpaired t test. *P<0.05 **P<0.01, ***P<0.001, ****P<0.0001.

Macrophages and dendritic cells are involved in tumor recognition and initiation of inflammation, and do so by expressing pattern recognition receptors (PRRs) that recognize different signals such as danger-associated molecular patterns (DAMPs) (26). To evaluate the functionality of the human myeloid cells in this model, we began by confirming that these cells expressed different TLRs at steady state, such as TLR4 and TLR8 (**Supplementary Figure 4**). As the innate immune response in this model can derive from both mouse and human cells, we decided to address the functionality of human innate immune cells in an *in vitro* setting, which enables the differentiation between human and murine cells. Functionality of TLRs was analyzed by treating human monocytes (hCD14^+^) isolated from hFlt3L-treated genO-BRGSF-HIS mice with LPS or Resiquimod (R848) and measuring cell activation. TLR4 and TLR7/TLR8 stimulation with LPS or R848 resulted in a significant increase in the frequency of human monocytes expressing activation receptors (human CD83) in contrast to unstimulated cells, showing that these innate immune recognition pathways are functional (**Figure 4A**). To analyze if TLR engagement would result in the secretion of different cytokines, human cells were isolated from the spleens of hFlt3L-boosted mice and treated with either poly(I:C) for TLR3 activation, R837 for TLR7 activation, R848 for TLR7/8 activation or CpG ODN for TLR9 activation. As expected, each compound led to the production of different human cytokines, an effect that was not observed upon treatment with RPMI alone, showing that these receptors are functional and can induce cytokine secretion upon activation (**Figure 4B**). *In vivo* stimulation of hFlt3L-boosted genO-BRGSF-HIS mice with LPS, but not with PBS, significantly increased the serum concentration of hIL-6 (~5000-fold) and hTNF-α (~2000-fold), confirming the activation and functionality of TLR4 (**Figure 4C**) in human cells *in vivo*. TREM1 is another receptor that plays an important role in innate immunity by amplifying the inflammatory response through interaction with DAP12. To analyze the functionality of this receptor, bone marrow cells from hFlt3L-treated mice were stimulated with LPS alone, or LPS and a TREM1 agonist. Unstimulated bone marrow cells showed very low levels of cytokine production, whereas LPS treatment resulted in a significant increase of hIL-6, hTNF-α and hIL-8 production. However, treatment with both LPS and a human-specific TREM1 agonist led to a significantly higher human cytokine and chemokine secretion relative to LPS alone (**Figure 4D**), showing that this receptor is functional. Similarly, hCD45^+^ enriched splenocytes treated with 2’3’ cGAMP, a cyclic dinucleotide known to be a potent agonist of STING (27), showed production of hIFN-γ, hIFN-α2 and hIP-10, demonstrating that the STING signaling pathway is functional in the human cells derived from genO-BRGSF-HIS mice (**Figure 4E**). Furthermore, treatment with different STING agonists resulted in a dose-dependent production of hIL-6, hIL-1β and hTNF-α (data not shown). Similarly, 2’3’cGAMP treatment of human cells isolated from the spleens of hFlt3L-treated genO-BRGSF-HIS mice resulted in a significant increase in the frequency of CD69^+^ on T cells, further confirming the functionality of this pathway (**Supplementary Figure 5**). Taken together, these data demonstrate that the human myeloid cells of genO-BRGSF-HIS mice express different PRRs, and these receptors respond to different stimuli and induce their respective signaling pathways.

### 2.3 Immune cell infiltration in MDA-MB-231 tumors recapitulates the dynamic and complexity of the tumor microenvironment

**Figure 5:**
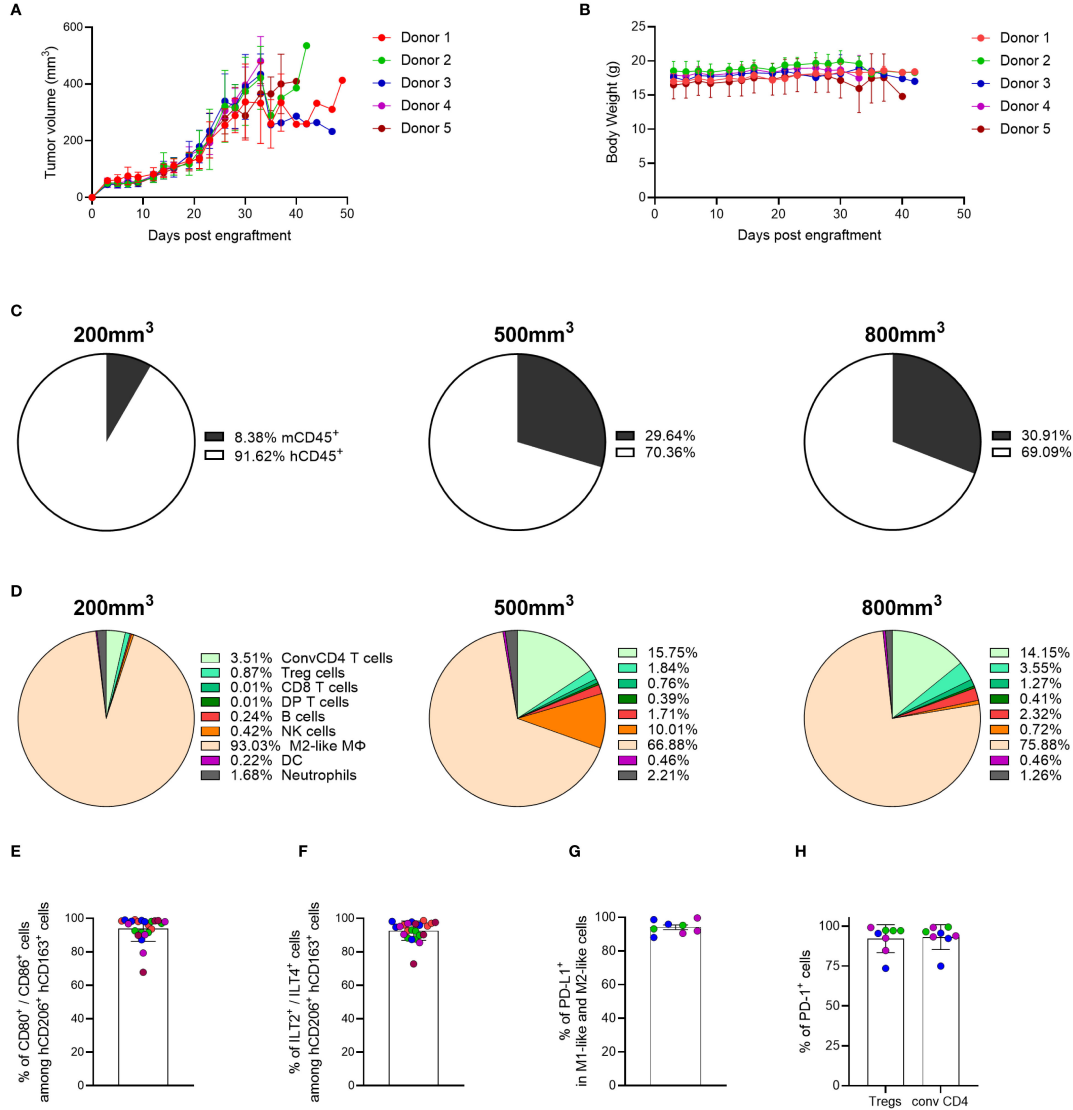
The MDA-MB-231 TME is mainly composed of myeloid cells, and its composition is dependent on tumor burden. **(A)** Tumor growth was measured over time in hFlt3L-treated genO-BRGSF-HIS mice engrafted with hCD34^+^ cells and implanted with 10 x 10^6^ MDA-MB-231 cells. **(B)** Mouse body weight was quantified following implantation with 10 x 10^6^ MDA-MB-231 cells in hFlt3L-treated genO-BRGSF-HIS mice. Mouse body weight is represented per hCD34^+^ HSC donor. For graphs **(A, B)**, five mice were used per donor (25 mice in total). **(C)** Quantification of the percentage of murine or human CD45^+^ cells as part of the immune cell infiltrate in MDA-MB-231 tumors at an average size of 200, 500 or 800 mm^3^. Mean percentages are plotted. **(D)** Quantification of the cell populations present in the human immune infiltrate of the MDA-MB-231 TME at an average size of 200, 500 or 800 mm^3^ by cytometry. Mean percentages are plotted. For **(C, D)**, the graphs represent tumors in 19 hFlt3L-treated genO-BRGSF-HIS mice, engrafted with hCD34^+^ cells from three donors and implanted with 10 x 10^6^ tumor cells. Six mice were used in the 200 mm^3^ timepoint (2 mice/donor), nine for the 500 mm^3^ timepoint (3 mice/donor) and four mice for the 800 mm^3^ timepoint (1 mouse for donors 1 and 2, two mice for donor 3). **(E)** Quantification of activated M2-like macrophages (CD80^+^/CD86^+^) within the M2-like population (CD206^+^/CD163^+^). **(F)** Percentage of cells expressing ILT2 and ILT4 among the M2-like macrophage population (CD206^+^/CD163^+^). Graphs **(E, F)** represent tumors in 24 hFlt3L-treated genO-BRGSF-HIS mice. **(G)** Percentage of cells expressing PD-L1 among the population of M1-like (CD206^-^/CD163^-^/CD86^+^) and M2-like macrophages (CD206^+^/CD163^+^). **(H)** Mean percentage of cells expressing PD-1 among the population of Tregs and conventional CD4 T cells in tumors of 400–600 mm^3^. Graphs **(G, H)** represent tumors in 8 hFlt3L-treated genO-BRGSF-HIS mice. In panels **(E–H)**, data was acquired in tumors of 400–600 mm^3^, individual points represent individual mice, and the different colors represent different donors (five in total for **E, F** and three for **G, H**). Error bars represent standard deviation.

Following *in vitro* assessment of the functionality of genO-BRGSF-HIS myeloid cells, we investigated whether these cells would also be functional *in vivo*. To investigate immune cell functionality and plasticity in response to tumor development, genO-BRGSF-HIS mice were engrafted with human breast cancer cells, MDA-MB-231, a tumor type that has been described to be highly infiltrated with myeloid cells (29). Mice received a single hFlt3L boost prior to tumor cell implantation and tumor analysis and tumor development revealed that all mice developed tumors upon engraftment, showing a take-rate of 100% (**Figure 5A**). Mice did not show clinical signs of illness, as evidenced by a constant body weight throughout the experiment (**Figure 5B**).

To analyze whether tumor cell composition and cell activation in the genO-BRGSF-HIS mice recapitulated the diversity reported in patient tumors, the composition of the MDA-MB-231 TME was investigated. Characterization of immune cell infiltration into the tumor was performed at three tumor-growth timepoints, averaging a size of 200, 500 or 800 mm^3^. Analysis of the cell infiltrate revealed that human cells represented over 90% of the immune cells infiltrated in the TME at the earliest timepoint, while they comprised approximately 70% of the immune cells at the intermediate and latest timepoints analyzed (**Figure 5C**). The distribution of immune cell populations in the TME also changed depending on the tumor size analyzed, with the MDA-MB-231 tumor being mainly composed of M2-like macrophages (CD206^+^/CD163^+^) (93% at 200 mm^3^ vs 76% at 800 mm^3^). An increase of conventional CD4^+^ T cells and NK cells was observed at later timepoints (3.5% of conventional CD4 T cells at 200 mm^3^ vs 14.5% at 800 mm^3^, and 0.42% of NK cells at 200 mm^3^ vs 10% at 500 mm^3^), highlighting the impact of tumor burden in TME composition (**Figure 5D**). Although the ratio of human-murine infiltration was similar among the mice reconstituted with different hCD34^+^ HSC donors (**Supplementary Figure 6A**), the differences in TME composition demonstrated some variation among different hCD34^+^ HSC donors (**Supplementary Figure 6B**). Given the high prevalence of myeloid cells in the TME of the MDA-MB-231 tumor and their role in modulating the immune environment of the TME, we further analyzed this cell population. Strikingly, most of the M2-like macrophages displayed the activated macrophage markers CD80^+^/CD86^+^ (**Figure 5E**) and expressed the human-specific receptors ILT2 and ILT4, further confirming the immunosuppressive environment of the TME (**Figure 5F**). This led us to interrogate whether the macrophages in the TME expressed PD-L1, a known T-cell inhibitory protein that promotes immune tolerance. As expected, cytometry analysis of M1-like (CD206^-^/CD163^-^/CD86^+^) and M2-like macrophages (CD206^+^/CD163^+^) demonstrated that almost 100% of these cells expressed PD-L1 (**Figure 5G**). Additionally, PD-1 was found to be expressed in almost 100% of the analyzed regulatory T cells and conventional CD4 T cells (**Figure 5H**), which might indicate activation/exhaustion of these cells within the TME. Altogether, these data show a dynamic composition of the tumor microenvironment in the genO-BRGSF-HIS mouse model, which is dependent on tumor burden, and an immune cell activation status that is characteristic of tumors. Importantly, myeloid cell development and recruitment to the tumor was observed following a single boost with hFlt3L. Additionally, the activation markers expressed on the tumor-associated macrophages (TAMs) further demonstrate that these cells are functional and capable of responding to environmental cues. The high percentages of myeloid cells present in the TME of MDA-MD-231 tumor suggest that this tumor model could be used for the assessment of myeloid-targeted therapies.

### 2.4 The TME of A549 tumors is dominated by human NK cells

**Figure 6:**
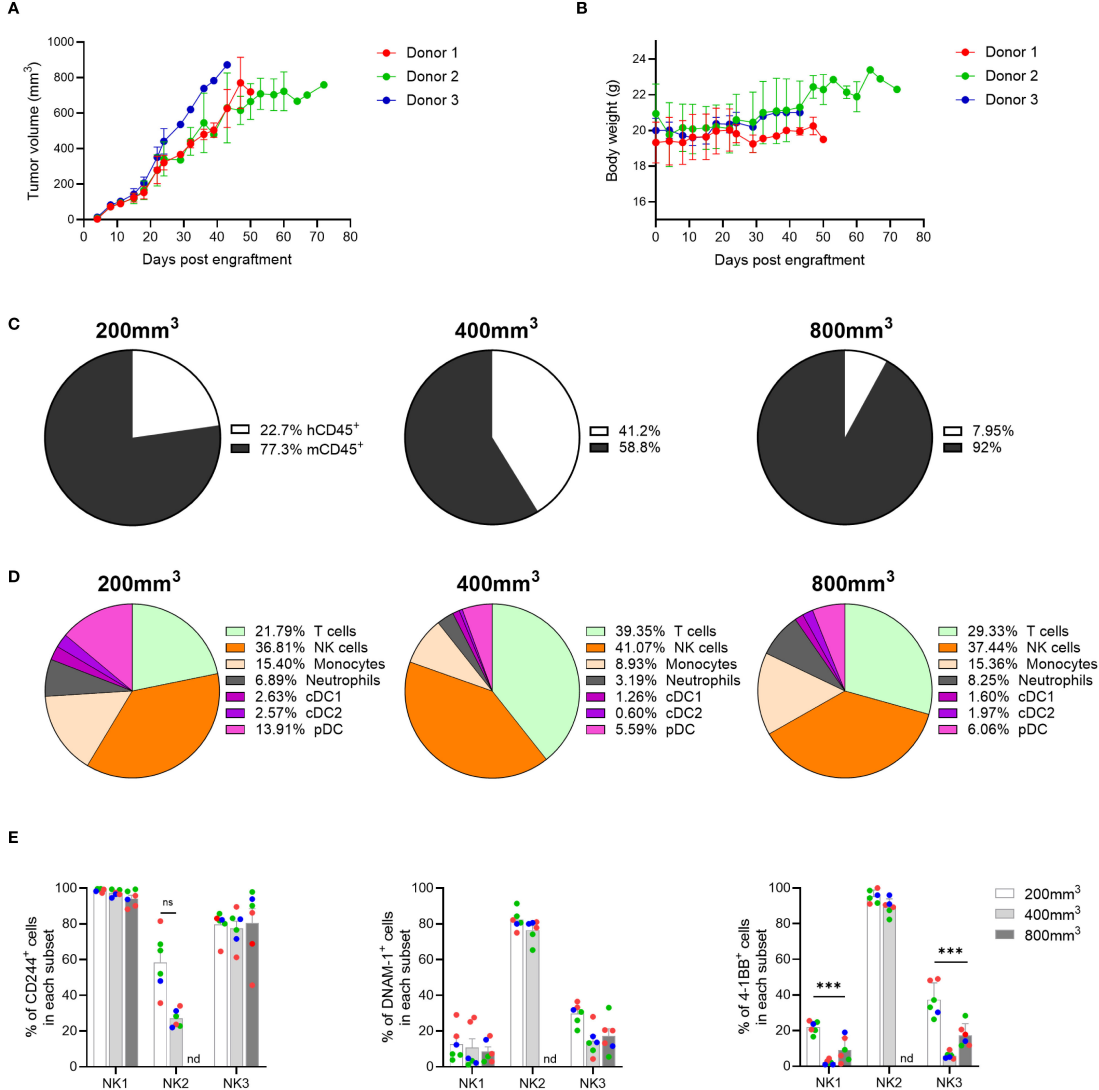
NK cells are recruited to the A549 TME and display activation markers. **(A)** Tumor growth was measured over time in genO-BRGSF-HIS mice engrafted with hCD34^+^ cells from three donors and implanted with 5 x 10^6^ A549 cells. **(B)** Quantification of mouse body weight represented per hCD34^+^ HSC donor. For graphs **(A, B)**, seven mice were used for donors 1 and 2, and four mice for donor 3 (18 mice in total). **(C)** Quantification of the percentage of murine or human CD45^+^ cells as part of the immune cell infiltrate in A549 tumors at an average size of 200, 400 or 800 mm^3^. Mean percentages are plotted. **(D)** Quantification of the cell populations present in the human immune infiltrate of the A549 TME at an average size of 200, 500 or 800 mm^3^ by cytometry. Mean percentages are plotted. For **(C, D)**, the graphs represent tumors in six genO-BRGSF-HIS mice per timepoint, engrafted with hCD34^+^ cells from three donors and implanted with 5 x 10^6^ tumor cells. In the 200 mm^3^ timepoint, 3 mice were used for donor 1, 2 mice for donor 2 and 1 mouse for donor 3. In the 500 mm^3^ timepoint, 2 mice/donor were used. In the 800 mm^3^ timepoint, 2 mice were used for donor 1, 3 mice for donor 2 and 1 mouse for donor 3. **(E)** Quantification of the percentage of different NK cells expressing activation markers, in 200, 400 or 800 mm^3^ tumors. nd, not detected. Individual points represent individual mice and the different colors represent different donors (three in total). Error bars represent standard error of the mean. Data were analyzed with a two-way ANOVA. ***P<0.001.

Given the diversity of immune cells present in the TME of MDA-MB-231 tumors, we questioned whether the composition of the TME in this mouse model would also significantly change upon implantation with different tumor cell lines, allowing the testing of immunotherapies targeting different cell populations. In recent years, NK-targeted therapies have received much attention due to their role in tumor elimination and immune surveillance, leading us for interrogate whether the genO-BRGSF-HIS mice would be suitable for testing such therapies. Since the A549 cell line, a tumor model of human lung carcinoma, has been reported to be highly infiltrated by NK cells (29), we employed this cell line to investigate the TME composition in this mouse model. As such, genO-BRGSF-HIS mice were implanted with A549 cells, and tumor growth and tumor composition were analyzed. The genO-BRGSF-HIS mice were permissive to implantation with these cells, as all mice developed tumors (**Figure 6A**) with no associated clinical signs, and maintained a constant body weight throughout the study (**Figure 6B**). Characterization of immune cell infiltration showed that this tumor type was poorly infiltrated by immune cells, irrespective of the tumor size analyzed (**Supplementary Figure 7A**), and that the humanization rate changed between hCD34^+^ HSC donors (**Supplementary Figure 7B**). Within the infiltrated immune cells, a high variation was observed between timepoints regarding the percentage of immune cells of human origin, with tumors of ~400 mm^3^ displaying the highest amount of human immune cells (41%) (**Figure 6C**). Analysis of the different cell populations that comprised the human cell infiltrate revealed that the TME of this tumor was dominated by human NK cells, which was observed in all three tumor sizes (**Figure 6D**). This was particularly striking, given the low percentage of NK cells detected in the blood of naïve, non-hFlt3L stimulated genO-BRGSF-HIS mice. Nonetheless, the TME composition of this tumor was still observed to be dynamic, as evidenced by the fluctuations in the percentages of all the cell populations analyzed. Given the high percentage of NK cells in the TME, and following the recent clustering and classification of three different NK subsets in the TME (30), we investigated whether these subsets could be detected. Indeed, NK1, NK2 and NK3 cells were detected in the TME (**Supplementary Figure 8**) and displayed an heterogeneous expression of the NK cell markers NKp30, NKp46, and CD16 at different timepoints (**Supplementary Figure 9**). Analysis of expression markers on NK2 cells at 800 mm^3^ could not be performed due to a very limited number of NK2 cells present at this timepoint. The activation status of the different populations was subsequently analyzed by monitoring the expression of CD244, DNAM-1 and 4-1BB, three NK cell activating receptors identified as good targets for cancer immunotherapies (31). All three markers were observed in the three populations (**Figure 6E**), showing the development and activation of NK cells in the genO-BRGSF-HIS model without the administration of exogenous human IL-15. In conclusion, we show that despite the low infiltration of immune cells, a high percentage of NK cells are recruited to the TME of A549 tumors, and that the three subsets of NK cells are detected within this cell population. Additionally, for this analysis, mice were not pre-treated with hFlt3L, showing that the tumor alone is enough to stimulate the production and recruitment of the myeloid cells detected in the TME of A549 tumors. The presence of these three different subsets of NK cells shows not only that this model reproduces what is seen in human biology (30) but that it can also be used to investigate human immunity, and for testing tailored therapeutics targeting different NK subsets.

### 2.5 The tumor microenvironment of HPAF-II tumors is mostly composed of T cells, enabling T-cell-targeted anti-cancer therapies

**Figure 7:**
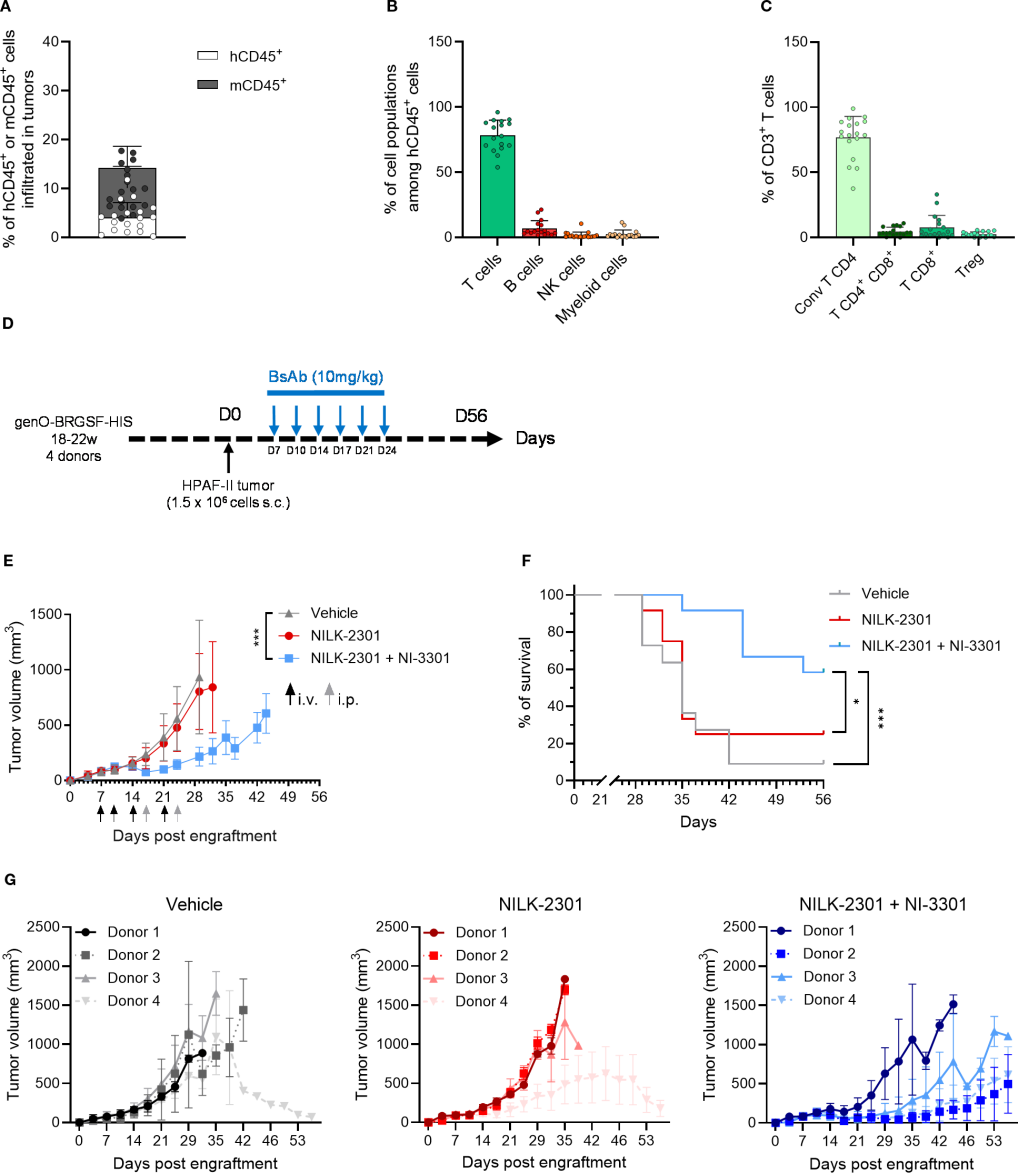
HPAF-II tumors in genO-BRGSF-HIS mice have a T-cell dominated TME and respond to T-cell engager combotherapy. **(A)** Immune cell infiltration was measured in the TME of HPAF-II tumors, and the percentage of murine or human CD45^+^ cells was calculated relative to the total cells of the tumors. **(B)** Quantification of the types of cells present in the human immune infiltrate of the HPAF-II TME by cytometry. **(C)** Quantification of the T cell subpopulations in TME of HPAF-II tumors by cytometry. The graphs in **(A–C)** represent tumors in 25 hFlt3L-treated genO-BRGSF-HIS mice implanted with 1.5 x 10^6^ HPAF-II cells. Individual points represent individual mice. **(D)** Scheme illustrating the experimental setup for tumor implantation and treatment with bispecific antibodies. **(E)** Tumor volume was followed over time in genO-BRGSF-HIS mice treated in monotherapy with the NILK-2301 bispecific antibody targeting CD3 and CEACAM5, or in combotherapy using NILK-2301 and NI-3301, which targets CD28 and CEACAM5. The graph represents 12 genO-BRGSF-HIS mice, engrafted with hCD34^+^ cells from four donors (three mice/donor). Statistical analysis performed on day 29 post engraftment between vehicle and NILK-2301 + NI-3301. Data were analyzed using a Mann Whitney test. i.v.- intravenous injection; i.p.- intraperitoneal injection. **(F)** Survival curves of genO-BRGSF-HIS mice treated with bispecific antibodies in mono- or combotherapy. Data were analyzed with a Log-rank (Mantel-Cox) test. **(G)** Individual tumor growth graphs represented by each treatment condition, displaying the tumor growth curves for each group of mice based on the hCD34^+^ HSC donor. Error bars represent standard deviation. *P<0.05, ***P<0.001.

Among the many therapies targeting different cell types, T-cell engagers (TCEs) have shown great promise in cancer treatment, with several approved TCE therapies already on the market. However, new TCEs targeting different types of cancer and with improved properties are still being developed, so preclinical models are needed to test these therapeutics. TCEs were recently shown to efficiently decrease HPAF-II tumor growth in genO-BRGSF-HIS mice (32), suggesting that this pancreatic adenocarcinoma tumor model is infiltrated by T cells. To analyze the TME composition of this tumor model, genO-BRGSF-HIS mice were implanted with HPAF-II cells. Mice were shown to be permissive to implantation with this cell line, as a 100% take-rate was observed (**Supplementary Figure 10A**). Although no body-weight loss was observed in tumor-bearing mice (**Supplementary Figure 10B**), most mice displayed tumor necrosis from day 30 onward (23 out of 25 mice), leading to early study termination, a phenomenon also observed by others (32). Analysis of the composition of the HPAF-II TME revealed a percentage of immune infiltration of around 15%, with about 5% of the infiltrated immune cells being of human origin (**Figure 7A**). Among these cells, about 80% were T cells (**Figure 7B**), mainly conventional CD4 effector T cells (**Figure 7C**). No striking difference was observed in the percentage of human immune infiltrate or cell subset composition between different hCD34^+^ HSC donors (**Supplementary Figures 11A, B**). Since the immune cell composition in the TME was dominated by T cells, we tested T-cell engagers (TCEs), either as monotherapy or as combination therapy in mice implanted with HPAF-II cells (**Figure 7D**). Mice were treated with NILK-2301 and NI-3301, two TCEs that target either CD3 (33) or CD28 (32), respectively, and CEA cell adhesion molecule 5 (CEACAM5), and tumor growth and mouse survival were followed for 56 days. When used as monotherapy, NILK-2301 was not sufficient for reducing tumor growth (**Figure 7E**). However, NILK-2301 administered in combination with NI-3301 resulted in a significant reduction in tumor growth (**Figure 7E**), which in turn resulted in a significant improvement of mouse survival (**Figure 7F**). Interestingly, the response to TCE treatment varied among different hCD34^+^ HSC donors (**Figure 7G**), reproducing the variability in response to treatment reported in patients (34). In accordance with previous studies (32), these results show that the genO-BRGSF-HIS model is a relevant model for testing the efficacy of immunotherapies targeting the lymphoid compartment, where a reduction of tumor growth is observed, despite the low rate of immune cell infiltration in this tumor. We confirm that the genO-BRGSF-HIS model is permissive to engraftment of different tumor cell lines, displaying high tumor uptake rates regardless of the tumor cell line employed and enabling a wide therapeutic window. The dynamic TME composition and the response to TCE therapy further demonstrate that this model is a useful tool for the study of tumor biology and the development of translational anti-tumor therapies.

## 3 Discussion

The development of immunocompetent mice with a human immune system has led to a leap in our understanding of cancer biology and its interaction with the immune system. These models have also enabled the preclinical testing of new cancer immunotherapies with a much greater degree of translatability and, consequently, success (3). Despite their advantage to investigate the human immune response in mice, HIS models have different limitations that could bias the interpretation of the results. This work therefore aimed at investigating the influence of the tumor type and tumor burden on human immune cell recruitment, activation, and differentiation in the genO-BRGSF-HIS model. Here we show that this mouse model features functional lymphoid and myeloid compartments, and that these cells are recruited to the TME of different tumors, where they express markers of polarization and/or activation. Importantly, the composition of the TME is very dynamic, being dependent on tumor type and tumor burden, reflecting the complexity and heterogeneity that is observed in patients (35–37), and showing that this could be a translational model for tumor biology studies. These data also suggest that this model is a relevant tool for efficacy testing of immunotherapeutics, enabling the analysis of compounds targeting different cell populations. Additionally, we show that human CD34^+^ HSC engraftment is stable for more than 31 weeks, with no associated clinical signs, allowing for long-term studies.

As previously shown by us and others (12, 24, 38), hCD34^+^ cell engraftment in different immunodeficient mouse models results in an immune cell distribution that is significantly different from that of humans, with a predominance of B cells. In this study, a change in the ratio of B and T cells over time was also observed. This could be explained by different kinetics of T-cell and B-cell development in HIS mice (15, 20, 21, 39). The decrease in B cell percentages and total numbers may also be explained by a lack of environmental factors that allow B cell survival, or due to the clearance of the excessive numbers of B cells (40). Nonetheless, the B cells developed in the genO-BRGSF-HIS mice are capable of immunoglobulin class switching and of immunoglobulin production, as evidenced by the detection of IgG1, IgE and IgA levels of around 2000, 50 and 2000 ng/mL, respectively, in the serum of naïve humanized mice (data not shown). We also show that both lymphoid and myeloid cells are developed in this model, including γδ T cells. These cells were previously shown to be functional, as they respond to γδ T cell-activators in a similar way as human PBMCs from a healthy donor (41).

The human myeloid and dendritic cells in genO-BRGSF-HIS mice were also shown to respond to a second boost of hFlt3L, resulting in increased production of cells to levels similar to those observed after the first boost. This data shows that the myeloid and dendritic compartments can be boosted at specific times that would be more relevant to a given experimental design, as well as extending the experimental window. As expected, the lymphoid cells did not strongly respond to hFlt3L, while the NK cells did, as previously reported (21). This is likely explained by a higher number of dendritic cells following hFlt3L treatment, resulting in increased cytokine levels, such as IL-15, known to induce the proliferation of NK cells (42, 43). Functional analysis of the myeloid cells of the genO-BRGSF-HIS model was also performed, revealing their potential to crosstalk with innate immune pathways. Analysis of human TLR expression and receptor functionality in human myeloid cells demonstrated that these cells respond to various stimuli, as evidenced by expression of the activation marker hCD83 and by human cytokine production following treatment with LPS (TLR4 agonist), R848 (TLR7/8 agonist), CpG ODN 2216 (TLR9 agonist); Poly(I:C) (TLR3 agonist), R837 (TLR7 agonist), a TREM1 agonist, or cGAMP (STING agonist). These *in vitro/ex vivo* studies allowed us to discriminate the response of human cells from the murine ones, as human cells were isolated before they were activated. *In vivo* LPS challenge showed the production of human cytokines, indicating that human cells were functional, however, murine cells also contribute somehow to this response, as LPS stimulates both human and murine TLR4. Moreover, previous analysis of the expression of Fcγ receptors by human immune cells in genO-BRGSF-HIS mice demonstrated that human FcγRI, FcγRII, and FcγRIII were expressed on the surface of both peritoneal macrophages (hCD45^+^ hCD3^-^ hCD19^-^ hCD14^+^) and BMDMs (hCD45^+^ hCD3^-^ hCD19^-^ hCD14^+^) (28).

Despite the differences in composition of genO-BRGSF-HIS blood and human blood, we show that myeloid, dendritic, and lymphoid cells are recruited into the TME in genO-BRGSF-HIS mice, and that one hFlt3L boost, or even none, is enough to observe this response. By analyzing the composition of the TME in three different cancer models—breast, lung and pancreatic adenocarcinoma—we observed a strikingly different composition of the immune cell infiltrate, consistent with what is reported for cancer patients (36, 37, 44). The TME of mice implanted with breast cancer cells MDA-MB-231 was primarily composed of myeloid cells, and predominantly of M2-like macrophages. In contrast, the TME of the lung cancer model, A549, consisted mainly of NK cells, while the one from the pancreatic cancer model, HPAF-II, was predominantly composed of T cells. In both the TME of MDA-MB-231 and A549 tumors, we detected activation markers expressed on the infiltrated immune cells. Most of M2-like macrophages in MDA-MB-231 tumors expressed CD80 and CD86, and over 90% of T cells expressed PD-1. Additionally, we could detect the three subsets of NK cells in the TME of A549 tumors, which expressed the activation markers CD244, DNAM-1 and 4-1BB. This suggests that the immune cells developed in the genO-BRGSF-HIS respond and adapt to different stimuli and tumor types, indicating that this model could be a useful and translational tool for studying tumor biology and testing targeted immunotherapies. In this context, functional assays should be performed to establish a causal relationship between immune cell infiltration and tumor control. Nevertheless, these data show human myeloid cell recruitment, activation, regulation and differentiation in the TME. Furthermore, they established significant development, recruitment and polarization of NK cells in the TME, although IL-15 was not supplemented exogenously nor by gene overexpression.

The characterization of the cell populations in the TME of different tumor types, as well as the markers they express, allows for the strategic and evidence-based development of new anti-cancer therapies. The demonstration of PD-L1 expression in tumor-associated macrophages, the suggested activation/exhaustion profile of T cells in the MDA-MB-231 tumor, or the identification of different NK cell subsets in the A549 tumor provide key examples for the development of targeted therapies. Blockade of the PD-1/PD-L1 axis has been shown to be a successful strategy to enhance T-cell responses in TBNC patients and ultimately improve treatment outcomes (45), as well as NK cell-directed therapies (46). As shown here, the genO-BRGSF-HIS mice provide useful insights into the cellular composition of different tumors. The observation that the TME of the HPAF-II pancreatic cancer model was composed mainly of lymphoid cells led to the investigation of the efficacy of new bispecific antibodies targeting T cells. Although these TCEs have not been tested in non-humanized genO-BRGSF mice, and thus the contribution of non-immune cells in their anti-tumor response cannot be ruled out, the Fc-domain of both TCEs has been modified to avoid binding to Fcγ receptors. Moreover, NILK-2301 and NI-3301 have been designed to drive T-cell retargeting to tumor cells and to provide the co-stimulatory response, respectively. Despite the reduced level of immune cell infiltration in HPAF-II tumors, a significant reduction in tumor burden and improvement in mouse survival were observed following TCE treatment. TME infiltration analyses in non-treated mice showed a predominance of conventional CD4 T cells, however, it remains to be investigated whether these cells are cytotoxic CD4 T cells or if a low number of infiltrated cytotoxic CD8 T cells are capable of mediating TCE response. Together with previous reports (32, 47, 48), the efficacy assessment of TCE in genO-BRGSF-HIS mice highlights the relevance of this model for cancer studies and testing of new therapies.

Furthermore, T-cell targeted therapies aiming at repolarizing the TME displayed promising results in the genO-BRGSF-HIS model (48) and additionally, this model has also been shown to be a useful tool for testing myeloid-targeted therapies, such as MACO355, which repolarizes M2 macrophages into a pro-inflammatory state by binding LILRB1, LILRB2 and LILRB3 (47). Besides the importance of cancer and immune cells for tumor growth and modulation, other factors, such as the tumor stroma, also strongly influence cancer development. Stromal cells secrete a plethora of signals such as growth factors, micro-RNAs, and cytokines that provide important cues for invasion, immune regulation, angiogenesis, and tumor growth. In genO-BRGSF-HIS mice, tumor and immune cells are of human origin, and stromal cells are of murine origin, resulting in a potential lack of cross-reactivity between murine signals and human receptors, or vice versa. This lack of complete cross-reactivity may therefore lead to a biased interpretation of tumor-immune cell interaction and drug response. The potential contribution of murine stroma in tumor development and immunotherapy assessment has not been investigated in genO-BRGSF-HIS mice. To circumvent this limitation in genO-BRGSF-HIS and other humanized mice, assessment of immunotherapies could be complemented with their assessment in immunocompetent mouse models, where the crosstalk among murine stroma, murine tumor and murine immune cells is fully functional.

A current drawback in the steps of preclinical evaluation of new therapies is the lack of available models to enable simultaneous assessment of efficacy and safety, making it difficult to correlate the results, and highlighting the need to be able to perform both efficacy and safety analysis in the same model. Previous studies have shown the relevance of the genO-BRGSF-HIS model for safety studies by testing for the onset of cytokine release syndrome (3, 24), which has been demonstrated with compounds targeting lymphoid (OKT3) or myeloid (SNS-101 and JNJ, two VISTA-targeting constructs) cells, demonstrating the versatility of this model. However, as the genO-BRGSF-HIS model displays a correct expression pattern of different targets, including CCR8, known to be differentially expressed between tumors and the periphery (49), and between mice and humans, this model also enables the simultaneous evaluation of both efficacy and safety, opening up the possibility to evaluate different types of immunotherapies. Additionally, since this model contains a human immune system, and the immune cells contain the human genomic sequence, this model could also enable the testing of therapeutic antisense oligonucleotides. This new type of therapy can be used to precisely control the expression of genes involved in tumor growth, immune evasion, and even therapy resistance (50).

The ability to thoroughly characterize the cell populations present in the TME is of particular importance due to the potential to develop new anticancer strategies such as 1) reprogramming of cells to an pro-inflammatory, anti-cancer phenotype; 2) development of precisely targeted anticancer therapies, either by targeting a specific cell type or a specific protein, such as an immune checkpoint; 3) prediction of cancer and treatment outcome; and 4) identification of new biomarkers that would allow the development of new therapies and stratification strategies. The development of both myeloid and lymphoid compartments makes this model a useful tool for studying human immune responses in the context of disease or not, as well as allowing the evaluation of therapies targeting either lymphoid or myeloid cells.

## 4 Materials and methods

### 4.1 genO-BRGSF-HIS mice

Newborn BALB/c Rag2^tm1Fwa^Il2rγ_c_^tm1Cgn^Sirpα^NOD^Flt3^tm1lrl^ mice were reconstituted with a human immune system (HIS) following full body irradiation conditioning (2.8 Gy; X-ray source), by intrahepatic injection of ~1x10^5^ human hematopoietic progenitor cells (hCD34^+^ cord blood cells) (purchased from Lonza (USA) and CTI Biotech (France)). The humanization rate was analyzed 12 weeks later by flow cytometry. Only mice displaying over 30% of humanization (percentage of human CD45^+^ cells in total number of human and mouse CD45^+^ cells) were included in the experiments. For all experiments, mice were randomized within groups according to donor and tumor size when applied, in order to ensure comparable tumor sizes and to include different donors in each group. Mice were housed in ventilated cages with EOPS sanitary status (19–23°C, 30–70% humidity, 12-hour night/day cycle), and provided with *ad libitum* access to sterile water and autoclaved feed (SAFE^®^). For studies involving tumor development, genO-BRGSF-HIS mice were inoculated with tumor cells when they were 16–22 weeks of age. For other studies, experiments were also performed when mice were 16–22 weeks old, unless stated otherwise. The experiments involving the use of human cells for the generation of humanized mice were approved by an ethical committee (VetAgro Sup n°018) and validated by the French Ministry of Education and Research (APAFIS#30015). All other animal experimental procedures were ethically approved by the ethical committee VetAgro Sup n°018, validated by the French Ministry of Education and Research (APAFiS #38722) and performed in line with relevant guidelines and regulations including the EU Directive 2010/63/EU, the related French décret n° 2013-118, and the 3Rs (Refinement, Reduction, Replacement).

### 4.2 Animal treatments

genO-BRGSF-HIS mice received four intraperitoneal injections every two to three days (D0, D2, D4 and D7) of 10 µg (in 150 µL of PBS 1x) of recombinant human Flt3L (recombinant Flt3L-Ig (hum/hum), BioXcell, cat. BE0098). For experiments in which mice received a second boost of hFlt3L, genO-BRGSF-HIS mice received another four intraperitoneal injections every two to three days (D21, D23, D25 and D28) of 10 µg of recombinant human Flt3L. For stimulation of TLR4, mice were intraperitoneally injected with LPS at 2 mg/kg (serotype O127:B8), one day after the final day of hFlt3L treatment. Blood was collected 4 hours later into EDTA-coated tubes by intracardiac punction for cytokine analysis. For efficacy assessment of bispecific antibodies (BsAb), mice were subcutaneously implanted with 1.5 x 10^6^ HPAF- II cells. Seven days later, mice were enrolled for randomization and assigned to treatment groups to ensure comparable tumor volume (TV) means and standard deviations between groups. Additionally, randomization was structured to include three mice per donor in each group, as individual donors may exhibit varying responses to therapy. Mice were then treated with 10 mg/kg of NILK-2301, a bsAb targeting CD3 and CEACAM5 (33), or a combination of NILK-2301 and NI-3301, which targets CD28 (32), and CEACAM5, or just with the vehicle solution. Mice received 6 treatments over the course of two weeks (every 3–4 days). The administrations were performed either intravenously or intraperitoneally, in order not to damage the injection areas. Tumor volume and mice survival were monitored every three to four days.

### 4.3 Clinical monitoring

Following randomization based on hCD34^+^ HSC donor and tumor size, body weight and mouse survival were measured at the indicated timepoints and initiation of treatment. Clinical signs were monitored and the observation of one of the following criteria for more than 24 hours led to the euthanasia of the mouse: body weight loss superior to 15%, stereotyped behavior, paralysis, severe lameness, hunched posture or prostration, tremor or tumor ulceration superior to 1.5 mm.

### 4.4 Cell line maintenance

Cells were cultured in RPMI 1640 supplemented with 10% FBS, 100 U/mL penicillin and 100 µg/mL streptomycin and incubated at the 37°C with 5% CO_2_. Cell treatments for DC and monocyte activation are described below.

MDA-MB-231, an adherent cell line originating from human breast adenocarcinoma, was purchased from ATCC (#HTB-26) and maintained according to manufacturer’s recommendations. A549, an adherent cell line originating from human lung carcinoma, was purchased from ATCC (#CCL-185) and maintained according to manufacturer’s instructions. HPAF-II cells, a human pancreatic adenocarcinoma adherent cell line, were purchased from ATCC (#CRL-1997) and maintained in EMEM supplemented with 10% of heat-inactivated complete fetal bovine serum, 2 mM L-Glutamine (Sigma-Aldrich, #G7513) and 1 mM sodium pyruvate (Sigma-Aldrich, #S8636).

Cells were sub-cultured upon reaching a maximum confluency of 80% during routine culture at 37°C in a humidified atmosphere containing 5% CO_2_.

### 4.5 Cell preparation for subcutaneous injection

On the day of injection, the cells were detached using 1x TrypLE. The cell suspension was gently homogenized and counted using trypan blue exclusion and a Malassez hemocytometer. Cells were washed once with HBSS to ensure complete FBS elimination, before being centrifuged for the second time. Cell pellet was resuspended in HBSS to obtain the desired concentration for injection. The day before injection, animals were shaved over their right flank. On the day of injection, mice were anesthetized with gaseous isoflurane and cells were subcutaneously injected into the flank.

### 4.6 Splenocyte and bone marrow activation assay

CD86 expression on monocytes was investigated using the spleens from 8 genO-BRGSF-HIS mice treated with hFLt3L. Spleens were collected one day after the final treatment with hFLt3L (3 hCD34^+^ cell donors were used) and processed as described below, and the cell population was enriched by positive selection for hCD14 and hCD123 using hCD14 MicroBeads (Miltenyi, #130-050-201) and hCD123 MicroBeads (Miltenyi, #130-094-432) and by exclusion of mCD45, mTer119, hCD3, hCD19 and hCD20. Upon enrichment and mCD45 cell depletion, 1-5 x 10^5^ splenocytes were stimulated for 24h with 250 µg/mL of R848 (Resiquimod, Invivogen, #tlrl-r848) or LPS at 0.1 µg/mL (for stimulation of TLR4, Sigma, #L4516).

For cytokine measurements, bone marrow cells or human cells isolated from splenocytes were isolated from 4-9 genO-BRGSF-HIS mice treated with hFLt3L. For splenocyte stimulation, mouse CD45^+^ cells were depleted and hCD45^+^-enriched cells were then stimulated with either CpG ODN 2216 (Invivogen #tlrl-2216) at 10 µM (for stimulation of TLR9); Poly(I:C) (Invivogen # tlrl-picw) at 10 µg/mL (for stimulation of TLR3), R837 (Invivogen # tlrl-imqs) at 10 µg/mL (for stimulation of TLR7); R848 (Resiquimod, Invivogen, #tlrl-r848) at 10 µg/mL (for stimulation of TLR7/8) or 2’3’ cGAMP (Invivogen # tlrl-nacga23) at 10 µg/mL (for stimulation of STING). Bone marrow cells were activated with 0.1 μg/mL LPS or 0.1 μg/mL LPS and a TREM1 agonistic antibody (10 μg/mL) or left unstimulated. Depending on the assay, 6 or 24 hours later, the cytokine secretion and cell activation was analyzed by cytometry, by looking at the specific cytokines or at the percentage of CD83^+^ CD14^+^ cells or CD69^+^ CD3^+^ cells.

### 4.7 Blood sampling and serum preparation

For cytometry analysis, whole blood samples (150 µL) were obtained via jugular vein puncture under gaseous anesthesia (isoflurane) and harvested in EDTA-coated microtubes (Microvette^®^ CB300 K2E). Flow cytometry analysis of blood cells was performed after leukocyte purification on a Ficoll density gradient. The percentage of human hematopoietic cells (hCD45^+^) was calculated among total live leukocytes, using the following formula: Humanization = (100 x %hCD45^+^)/(%mCD45^+^ + %hCD45^+^).

For cytokine analysis, at least 150 µL of whole blood per timepoint and per mouse was sampled through the jugular vein under gaseous anesthesia (isoflurane). Whole blood was kept at room temperature for one hour to allow coagulation, then centrifuged at 3000g for 10 minutes at 4°C. Supernatants containing sera were collected into new tubes and stored at -80°C until processed for cytokine analysis.

### 4.8 Cytokine quantification

Blood or cell culture media were collected at the indicated timepoints and kept on ice for processing. Blood was processed as described above for serum collection. Supernatant was centrifuged at 4°C for 5 minutes, at 500g. Cytokine analysis was performed using LEGENDplex™ Human Anti-Virus Response Panel kit (Biolegend, #740390), according to manufacturer’s instructions, and detected using a Attune NxT Flow Cytometer or ID7000 Spectral Cell Analyzer (Sony Biotechnology).

### 4.9 Spleen, bone marrow and tumor processing for flow cytometry analysis

Mice were anesthetized with isoflurane followed by final sacrifice using cervical dislocation. Spleens were harvested in RPMI 1640 medium with 1% penicillin-streptomycin and digested using the Spleen Dissociation Kit and GentleMACS Octo Dissociator with Heaters (Miltenyi Biotec) per manufacturer’s instructions.

Tumors were collected in RPMI 1640 medium with 1% penicillin-streptomycin and then digested using the Tumor Dissociation Kit, (Miltenyi Biotec; #130-095-929) and GentleMACS Octo Dissociator with Heaters (Miltenyi Biotec), according to manufacturer’s instructions. Undigested tissues and debris were removed by filtering the cellular solution through a 70 µm filter in 1x-PBS + 3% FBS. Cell number and viability were evaluated using a Luna-FLTM automated cell counter. One million (1 x 10^6^) cells were used for flow cytometry analysis.

Bone marrow cells were isolated from hFlt3L-treated genO-BRGSF-HIS mice following a standard protocol (51).

### 4.10 Depletion of mouse CD45^+^ cells

Splenocytes were resuspended in FACS Buffer and incubated with FcR Blocking Reagent (dilution 1/3) and anti-mCD45 biotin conjugated antibody (dilution 1/4) at 4°C for 5 minutes. Cells were then incubated with Anti-Biotin MicroBeads following manufacturer’s instructions. Unlabeled human cells were collected upon magnetic sorting using AutoMACS (Miltenyi Biotec).

### 4.11 Cytometry for immunoprofiling and humanization rates

Cells were incubated with Live/Dead Fixable Aqua (Dead Cell Stain kit; ThermoFisher, #L34957) for 20 minutes at 4°C. They were then washed once in 1x-PBS + 3% FBS and cell pellets were resuspended in 100 µL of the specific antibody cocktail mix (list of antibodies used in **Supplementary Table 1**) in the presence of Fc-blocking reagent (Miltenyi, #130-059-901 and #130-029-575) targeting murine Fc receptors and incubated for 20 min at 4°C in the dark. Cells were washed in FACS Buffer (PBS 1x, 3% FBS, 2mM EDTA) before flow cytometry acquisition (ID7000 Spectral Cell Analyzer, Sony Biotechnology). A threshold of a minimum of 50 cells per gate was used for proceeding with the analysis.

### 4.12 Tumor growth

genO-BRGSF-HIS mice were treated with hFlt3L as described above. One day after the final treatment with hFlt3L, mice were subcutaneously implanted with 1.5 x 10^6^ HPAF-II cells/mouse, 10 x 10^6^ MDA-MB-231 cells/mouse or with 5 x 10^6^ A549 cells/mouse. Tumor size and mouse body weight were monitored every 2–3 days. For experiments where the tumor microenvironment was analyzed, mice were sacrificed at the specific timepoints and tumors were harvested into RPMI 1640 medium with 1% penicillin-streptomycin and processed as described for cytometry analysis.

For tumor growth studies following treatment with bispecific antibodies, genO-BRGSF-HIS mice were not treated with hFlt3L.

### 4.13 Statistical analyses

Quantification and statistical analysis were performed using Excel and GraphPad Prism. The numerical data are presented as means ± SD or SEM when indicated. The differences were determined by one-way ANOVA, two-way ANOVA Multiple comparison, unpaired t-test, Welch t-test or by a Mann Whitney test as stated in the figure legends. Survival data were analyzed with a Log-rank (Mantel-Cox) test. P value < 0.05 was considered statistically significant (*). *P<0.05 **P<0.01, ***P<0.001, ****P<0.0001. All studies were unblinded.

## Data Availability

The original contributions presented in the study are included in the article/**Supplementary Material**. Further inquiries can be directed to the corresponding author.

## References

[1] Iadonato S Ovechkina Y Lustig K Cross J Eyde N Frazier E . A highly potent anti-VISTA antibody KVA12123 - a new immune checkpoint inhibitor and a promising therapy against poorly immunogenic tumors. Front Immunol. (2023) 14:1311658. doi: 10.3389/fimmu.2023.1311658, PMID: 38152397 PMC10751915

[2] Johnston RJ Su LJ Pinckney J Critton D Boyer E Krishnakumar A . VISTA is an acidic pH-selective ligand for PSGL-1. Nature. (2019) 574:565–70. doi: 10.1038/s41586-019-1674-5, PMID: 31645726

[3] Thisted T Smith FD Mukherjee A Kleschenko Y Feng F Jiang Z-G . VISTA checkpoint inhibition by pH-selective antibody SNS-101 with optimized safety and pharmacokinetic profiles enhances PD-1 response. Nat Commun. (2024) 15:2917. doi: 10.1038/s41467-024-47256-x, PMID: 38575562 PMC10995192

[4] Currlin S Rivera A Ataya M D’Andrea J Humphrey A Ehrhart J . Nilogen oncosystems’s 3D-PREDICT tumoroid model is an ex vivo precision oncology and co-clinical trial enrichment platform. In: Regular and Young Investigator Award Abstracts. Hoboken, USA: BMJ Publishing Group Ltd (2023). doi: 10.1136/jitc-2023-SITC2023.0002

[5] Panchal A Seto P Wall R Hillier BJ Zhu Y Krakow J . COBRA™: a highly potent conditionally active T cell engager engineered for the treatment of solid tumors. mAbs. (2020) 12:1792130. doi: 10.1080/19420862.2020.1792130, PMID: 32684124 PMC7531513

[6] De La Rochere P Guil-Luna S Decaudin D Azar G Sidhu SS Piaggio E . Humanized mice for the study of immuno-oncology. Trends Immunol. (2018) 39:748–63. doi: 10.1016/j.it.2018.07.001, PMID: 30077656

[7] Chuprin J Buettner H Seedhom MO Greiner DL Keck JG Ishikawa F . Humanized mouse models for immuno-oncology research. Nat Rev Clin Oncol. (2023) 20:192–206. doi: 10.1038/s41571-022-00721-2, PMID: 36635480 PMC10593256

[8] Mian SA Anjos-Afonso F Bonnet D . Advances in human immune system mouse models for studying human hematopoiesis and cancer immunotherapy. Front Immunol. (2021) 11:619236. doi: 10.3389/fimmu.2020.619236, PMID: 33603749 PMC7884350

[9] Chen A Neuwirth I Herndler-Brandstetter D . Modeling the tumor microenvironment and cancer immunotherapy in next-generation humanized mice. Cancers. (2023) 15:2989. doi: 10.3390/cancers15112989, PMID: 37296949 PMC10251926

[10] Rongvaux A Willinger T Martinek J Strowig T Gearty SV Teichmann LL . Development and function of human innate immune cells in a humanized mouse model. Nat Biotechnol. (2014) 32:364–72. doi: 10.1038/nbt.2858, PMID: 24633240 PMC4017589

[11] Li Y Mention J Court N Masse-Ranson G Toubert A Spits H . A novel Flt3-deficient HIS mouse model with selective enhancement of human DC development. Eur J Immunol. (2016) 46:1291–9. doi: 10.1002/eji.201546132, PMID: 26865269

[12] Maser I-P Hoves S Bayer C Heidkamp G Nimmerjahn F Eckmann J . The tumor milieu promotes functional human tumor-resident plasmacytoid dendritic cells in humanized mouse models. Front Immunol. (2020) 11:2082. doi: 10.3389/fimmu.2020.02082, PMID: 33013879 PMC7507800

[13] Willis E Verrelle J Banerjee E Assenmacher C-A Tarrant JC Skuli N . Humanization with CD34-positive hematopoietic stem cells in NOG-EXL mice results in improved long-term survival and less severe myeloid cell hyperactivation phenotype relative to NSG-SGM3 mice. Vet Pathol. (2024) 61:664–74. doi: 10.1177/03009858231222216, PMID: 38197423 PMC11264550

[14] Janke LJ Imai DM Tillman H Doty R Hoenerhoff MJ Xu JJ . Development of mast cell and eosinophil hyperplasia and HLH/MAS-like disease in NSG-SGM3 mice receiving human CD34+ Hematopoietic stem cells or patient-derived leukemia xenografts. Vet Pathol. (2021) 58:181–204. doi: 10.1177/0300985820970144, PMID: 33208054 PMC8414369

[15] Mu Y Ohno Y Mochizuki M Kawai K Goto M Ogura T . Human dendritic cell differentiation in hematopoietic stem cell-transplanted NOG hFLT3L Tg/mFlt3 KO humanized mice. Immunol Lett. (2024) 270:106943. doi: 10.1016/j.imlet.2024.106943, PMID: 39536946

[16] Legrand N Huntington ND Nagasawa M Bakker AQ Schotte R Strick-Marchand H . Functional CD47/signal regulatory protein alpha (SIRPα) interaction is required for optimal human T- and natural killer- (NK) cell homeostasis *in vivo* . Proc Natl Acad Sci. (2011) 108:13224–9. doi: 10.1073/pnas.1101398108, PMID: 21788504 PMC3156191

[17] Capasso A Lang J Pitts TM Jordan KR Lieu CH Davis SL . Characterization of immune responses to anti-PD-1 mono and combination immunotherapy in hematopoietic humanized mice implanted with tumor xenografts. J Immunother Cancer. (2019) 7:37. doi: 10.1186/s40425-019-0518-z, PMID: 30736857 PMC6368764

[18] Lang J Capasso A Jordan KR French JD Kar A Bagby SM . Development of an adrenocortical cancer humanized mouse model to characterize anti-PD1 effects on tumor microenvironment. J Clin Endocrinol Metab. (2020) 105:26–42. doi: 10.1210/clinem/dgz014, PMID: 31513709 PMC7947837

[19] Tentler JJ Lang J Capasso A Kim DJ Benaim E Lee YB . RX-5902, a novel β-catenin modulator, potentiates the efficacy of immune checkpoint inhibitors in preclinical models of triple-negative breast Cancer. BMC Cancer. (2020) 20:1063. doi: 10.1186/s12885-020-07500-1, PMID: 33148223 PMC7641792

[20] Marín-Jiménez JA Capasso A Lewis MS Bagby SM Hartman SJ Shulman J . Testing cancer immunotherapy in a human immune system mouse model: correlating treatment responses to human chimerism, therapeutic variables and immune cell phenotypes. Front Immunol. (2021) 12:607282. doi: 10.3389/fimmu.2021.607282, PMID: 33854497 PMC8040953

[21] Lopez-Lastra S Masse-Ranson G Fiquet O Darche S Serafini N Li Y . A functional DC cross talk promotes human ILC homeostasis in humanized mice. Blood Adv. (2017) 1:601–14. doi: 10.1182/bloodadvances.2017004358, PMID: 29296702 PMC5728352

[22] Labarthe L Henriquez S Lambotte O Di Santo JP Le Grand R Pflumio F . Frontline Science: Exhaustion and senescence marker profiles on human T cells in BRGSF-A2 humanized mice resemble those in human samples. J Leukoc Biol. (2020) 107:27–42. doi: 10.1002/JLB.5HI1018-410RR, PMID: 31378988

[23] Bagaev A Kotlov N Nomie K Svekolkin V Gafurov A Isaeva O . Conserved pan-cancer microenvironment subtypes predict response to immunotherapy. Cancer Cell. (2021) 39:845–65.e7. doi: 10.1016/j.ccell.2021.04.014, PMID: 34019806

[24] Martin G Gonon A Martin-Jeantet P Renart-Depontieu F Biesova Z Cifuentes A . Myeloid and dendritic cells enhance therapeutics-induced cytokine release syndrome features in humanized BRGSF-HIS preclinical model. Front Immunol. (2024) 15:1357716. doi: 10.3389/fimmu.2024.1357716, PMID: 38384461 PMC10880010

[25] Christensen PKF Hansen AK Skov S Martel BC Larsen J Høyer-Hansen MH . Imiquimod induces skin inflammation in humanized BRGSF mice with limited human immune cell activity. Murayama MA editor PloS One. (2023) 18:e0281005. doi: 10.1371/journal.pone.0281005, PMID: 36800344 PMC9937455

[26] Lebegge E Arnouk SM Bardet PMR Kiss M Raes G Van Ginderachter JA . Innate immune defense mechanisms by myeloid cells that hamper cancer immunotherapy. Front Immunol. (2020) 11:1395. doi: 10.3389/fimmu.2020.01395, PMID: 32733461 PMC7363805

[27] Ablasser A Goldeck M Cavlar T Deimling T Witte G Röhl I . cGAS produces a 2′-5′-linked cyclic dinucleotide second messenger that activates STING. Nature. (2013) 498:380–4. doi: 10.1038/nature12306, PMID: 23722158 PMC4143541

[28] Renart-Depontieu F Martin G Moine V Burnet-Merlin C Creusat F Gonon A . Abstract 4139: Functional human myeloid cells in BRGSF-HIS humanized mice enables myeloid-directed therapy assessment. Cancer Res. (2023) 83:4139. doi: 10.1158/1538-7445.AM2023-4139

[29] Rios-Doria J Stevens C Maddage C Lasky K Koblish HK . Characterization of human cancer xenografts in humanized mice. J Immunother Cancer. (2020) 8:e000416. doi: 10.1136/jitc-2019-000416, PMID: 32217760 PMC7174072

[30] Rebuffet L Melsen JE Escalière B Basurto-Lozada D Bhandoola A Björkström NK . High-dimensional single-cell analysis of human natural killer cell heterogeneity. Nat Immunol. (2024) 25:1474–88. doi: 10.1038/s41590-024-01883-0, PMID: 38956378 PMC11291291

[31] Page A Chuvin N Valladeau-Guilemond J Depil S . Development of NK cell-based cancer immunotherapies through receptor engineering. Cell Mol Immunol. (2024) 21:315–31. doi: 10.1038/s41423-024-01145-x, PMID: 38443448 PMC10978891

[32] Majocchi S Lloveras P Nouveau L Legrand M Viandier A Malinge P . NI-3201 is a bispecific antibody mediating PD-L1–dependent CD28 co-stimulation on T cells for enhanced tumor control. Cancer Immunol Res. (2025) 13:365–383. doi: 10.1158/2326-6066.CIR-24-0298, PMID: 39760515 PMC11876958

[33] Seckinger A Majocchi S Moine V Nouveau L Ngoc H Daubeuf B . Development and characterization of NILK-2301, a novel CEACAM5xCD3 κλ bispecific antibody for immunotherapy of CEACAM5-expressing cancers. J Hematol OncolJ Hematol Oncol. (2023) 16:117. doi: 10.1186/s13045-023-01516-3, PMID: 38087365 PMC10717981

[34] Herpels M Ishihara J Sadanandam A . The clinical terrain of immunotherapies in heterogeneous pancreatic cancer: unravelling challenges and opportunities. J Pathol. (2023) 260:533–50. doi: 10.1002/path.6171, PMID: 37550956

[35] Group Young Researchers in Inflammatory Carcinogenesis Wandmacher AM Mehdorn A-S Sebens S . The heterogeneity of the tumor microenvironment as essential determinant of development, progression and therapy response of pancreatic cancer. Cancers. (2021) 13:4932. doi: 10.3390/cancers13194932, PMID: 34638420 PMC8508450

[36] Jackson HW Fischer JR Zanotelli VRT Ali HR Mechera R Soysal SD . The single-cell pathology landscape of breast cancer. Nature. (2020) 578:615–20. doi: 10.1038/s41586-019-1876-x, PMID: 31959985

[37] De Lucia A Mazzotti L Gaimari A Zurlo M Maltoni R Cerchione C . Non-small cell lung cancer and the tumor microenvironment: making headway from targeted therapies to advanced immunotherapy. Front Immunol. (2025) 16:1515748. doi: 10.3389/fimmu.2025.1515748, PMID: 39995659 PMC11847692

[38] Ding Y Wilkinson A Idris A Fancke B O’Keeffe M Khalil D . FLT3-ligand treatment of humanized mice results in the generation of large numbers of CD141+ and CD1c+ Dendritic cells *in vivo* . J Immunol. (2014) 192:1982–9. doi: 10.4049/jimmunol.1302391, PMID: 24453245

[39] Meraz IM Majidi M Meng F Shao R Ha MJ Neri S . An improved patient-derived xenograft humanized mouse model for evaluation of lung cancer immune responses. Cancer Immunol Res. (2019) 7:1267–79. doi: 10.1158/2326-6066.CIR-18-0874, PMID: 31186248 PMC7213862

[40] LeBien TW Tedder TF . B lymphocytes: how they develop and function. Blood. (2008) 112:1570–80. doi: 10.1182/blood-2008-02-078071, PMID: 18725575 PMC2518873

[41] Martin G Gonon A Sonego F Cherifi Y Thiam K . 877 Assessment of γδ T cell therapies in humanized mice. In: Regular and Young Investigator Award Abstracts. Journal for ImmunoTherapy of Cancer (2024). doi: 10.1136/jitc-2024-SITC2024.0877

[42] Guimond M Freud AG Mao HC Yu J Blaser BW Leong JW . *In vivo* role of Flt3 ligand and dendritic cells in NK cell homeostasis. J Immunol. (2010) 184:2769–75. doi: 10.4049/jimmunol.0900685, PMID: 20142363 PMC2924750

[43] Yu H Fehniger TA Fuchshuber P Thiel KS Vivier E Carson WE . Flt3 ligand promotes the generation of a distinct CD34+Human natural killer cell progenitor that responds to interleukin-15. Blood. (1998) 92:3647–57. doi: 10.1182/blood.V92.10.3647, PMID: 9808558

[44] Liudahl SM Betts CB Sivagnanam S Morales-Oyarvide V Da Silva A Yuan C . Leukocyte heterogeneity in pancreatic ductal adenocarcinoma: phenotypic and spatial features associated with clinical outcome. Cancer Discov. (2021) 11:2014–31. doi: 10.1158/2159-8290.cd-20-0841, PMID: 33727309 PMC8338775

[45] Wang Z You P Yang Z Xiao H Tang X Pan Y . PD-1/PD-L1 immune checkpoint inhibitors in the treatment of unresectable locally advanced or metastatic triple negative breast cancer: a meta-analysis on their efficacy and safety. BMC Cancer. (2024) 24:1339. doi: 10.1186/s12885-024-13105-9, PMID: 39478479 PMC11526542

[46] Myers JA Miller JS . Exploring the NK cell platform for cancer immunotherapy. Nat Rev Clin Oncol. (2021) 18:85–100. doi: 10.1038/s41571-020-0426-7, PMID: 32934330 PMC8316981

[47] Wicher KB Haneklaus M Lopez-Yrigoyen M Poindron A Rodriguez-Seoane C Fransen M . Abstract LB040: MACO-355, a unique pan-LILR monoclonal antibody for cancer therapy, re-programs and stimulates immuno-suppressive macrophages in a novel ligand-binding blocking independent manner. Cancer Res. (2024). doi: 10.1158/1538-7445.AM2024-LB040

[48] Cohen OI Rudnick-Glick S Azoulay IS Fine T Elyada E Radomir L . 800 Preclinical *in vivo* characterization of the anti-tumor activity of a non-blocking PD-1 antibody fused to attenuated IL-2. In: Regular and Young Investigator Award Abstracts. Hoboken, USA: BMJ Publishing Group Ltd (2024). p. A905–5. doi: 10.1136/jitc-2024-SITC2024.0800

[49] Weaver JD Stack EC Buggé JA Hu C McGrath L Mueller A . Differential expression of CCR8 in tumors versus normal tissue allows specific depletion of tumor-infiltrating T regulatory cells by GS-1811, a novel Fc-optimized anti-CCR8 antibody. OncoImmunology. (2022) 11:2141007. doi: 10.1080/2162402X.2022.2141007, PMID: 36352891 PMC9639568

[50] Dean NM Bennett CF . Antisense oligonucleotide-based therapeutics for cancer. Oncogene. (2003) 22:9087–96. doi: 10.1038/sj.onc.1207231, PMID: 14663487

[51] Gonçalves R Mosser DM . The isolation and characterization of murine macrophages. Curr Protoc Immunol. (2015) 111:14.1.1–14.1.16. doi: 10.1002/0471142735.im1401s111 PMC2834554 19016445

