# Supplementary material for: Tumor-dependent myeloid and lymphoid cell recruitment in genO-BRGSF-HIS mice: a novel tool for evaluating immunotherapies
Source: Front Immunol. 2025 Sep 17;16:1624724. doi: 10.3389/fimmu.2025.1624724 (PMC12484184; doi:10.3389/fimmu.2025.1624724)
Supplement: Supplementary file 1 [file Table1.docx]

**Supplementary Materials and Methods**

**Supplementary Table 1:** List of antibodies used for cytometry stainings.

| **Target** | **Fluorochrome** | **Clone** | **Reactivity** | **Manufacturer** | **Reference** |
| --- | --- | --- | --- | --- | --- |
| CD3 | Alexa Fluor 488 | UCHT1 | human | BD Bioscience | #557694 |
| CD3 | BUV563 | OKT3 | human | BD Bioscience | #750974 |
| CD4 | Alexa Fluor 700 | RPA-T4 | human | Biolegend | #300526 |
| CD4 | BV650 | OKT4 | human | Biolegend | #317436 |
| CD8α | PE/Dazzle 594 | RPA-T8 | human | Biolegend | #301058 |
| CD11b | FITC | M1/70 | human | Biolegend | #101205 |
| CD11c | PE/Cyanine 7 | Bu15 | human | Biolegend | #337216 |
| CD11c | VioBright R720 | REA618 | human | Milteny | #130-128-247 |
| CD14 | Vioblue | REA599 | human | Milteny | #130-110-524 |
| CD14 | BV605 | M5E2 | human | Biolegend | #301833 |
| CD16 | BUV737 | 3G8 | human | BD Biosciences | #612786 |
| CD19 | PE-FIRE 700 | HIB19 | human | Biolegend | #302276 |
| CD33 | BV711 | WM53 | human | Biolegend | #303424 |
| CD33 | BUV615 | HIM3-4 | human | BD Biosciences | #752338 |
| CD45 | PerCP | HI30 | human | Biolegend | #304026 |
| CD45 | APC-efluor780 | 30-F11 | mouse | ThermoFischer Scientific | #47-0451-82 |
| CD45 | APC- FIRE750 | 30-F11 | mouse | Biolegend | #103154 |
| CD56 | PE | HCD56 | human | Biolegend | #318306 |
| CD56 | BV605 | HCD56 | human | Biolegend | #318334 |
| CD68 | BV785 | Y1/82A | human | Biolegend | #333826 |
| CD69 | PE | FN50 | human | Biolegend | #310906 |
| CD80 | PE-Dazzle594 | 2D10 | human | Biolegend | #305230 |
| CD80 | VioBright R720 |  | human | Miltenyi Biotec | #130-128-249 |
| CD86 | BV711 | IT2.2 | human | Biolegend | #305440 |
| CD86 | PE-Vio770 | REA968 | human | Miltenyi Biotec | #130-116-162 |
| CD123 | BB711 | 9F5 | human | BD Biosciences | #563161 |
| CD163 | PerCP-Vio700 | REA812 | human | Milteny | #130-112-133 |
| CD206 | BUV395 | 15-2 | human | BD Biosciences | #751778 |
| CD303 | BV421 | 201A | human | Biolegend | #354211 |
| TLR4 (CD284) | BV421 | HTA125 | human | Biolegend | #312811 |
| TLR8 (CD288) | APC | S16018A | human | Biolegend | #395506 |
| HLA-DR | APC | LN3 | human | Biolegend | #300424 |
| FcR-block |  |  | mouse | Miltenyi Biotec | #130-092-575 |
| FcR-block |  |  | human | Miltenyi Biotec | #130-059-901 |
| VSIG4 | APC | JAV4 | human | Life Technologies | # 17-5757-42 |
| PD-L1 | PE-Cy7 | MIH3 | human | Biolegend | # 374506 |
| PD1 | PE | EH12.2H7 | human | Biolegend | #BLE329906 |
| ILT-2 | APC | REA998 | Human | Miltenyi Biotec | #130-116-616 |
| ILT-4 | PE-Vio 615 | REA184 | human | Miltenyi Biotec | #130-129-366 |
| FoxP3 | APC | PCH101 | human | Invitrogen | #17-4776-42 |
| NKp46 | APC | 9E2 | human | Biolegend | #331918 |
| NKG2C | BV421 | 134591 | human | BD Biosciences | #748169 |
| NKp30 | PE-Dazzle594 | P30-15 | human | Biolegend | #325232 |
| NKG2D | BUV661 | 1D11 | human | BD Biosciences | #749932 |
| CD244 | Vio Bright B515 | REA112 | human | Miltenyi | #130-132-423 |
| NKG2A | BV711 | S19004C | human | Biolegend | #375156 |
| 4-1BB | PE | REA765 | human | Miltenyi | #130-110-763 |
| hDNAM-1 | PE-Cy7 | 11A8 | human | Biolegend | #338316 |
