# Supplementary material for: Tumor-dependent myeloid and lymphoid cell recruitment in genO-BRGSF-HIS mice: a novel tool for evaluating immunotherapies
Source: Front Immunol. 2025 Sep 17;16:1624724. doi: 10.3389/fimmu.2025.1624724 (PMC12484184; doi:10.3389/fimmu.2025.1624724)
Supplement: Supplementary file 2 [file DataSheet1.docx]

**Supplementary Figures**

**Supplementary Figure 1** – Total cell counts of humanization rate analysis, following engraftment with hCD34^+^ cells. **(A)** The rate of humanization was measured over 31 weeks (w) in genO-BRGSF-HIS mice (n=22) engrafted with hCD34^+^ cells from 2 donors (black circles and white circles), represented as the number of hCD45^+^ cells per microliter of blood. **(B)** Quantification of the different cell populations following hCD34^+^ HSC engraftment over 31 weeks, represented as absolute counts. Horizontal lines represent the mean. Data were analyzed using a Mann Whitney test. ***P<0.001, ****P<0.0001, ns – non-significant.


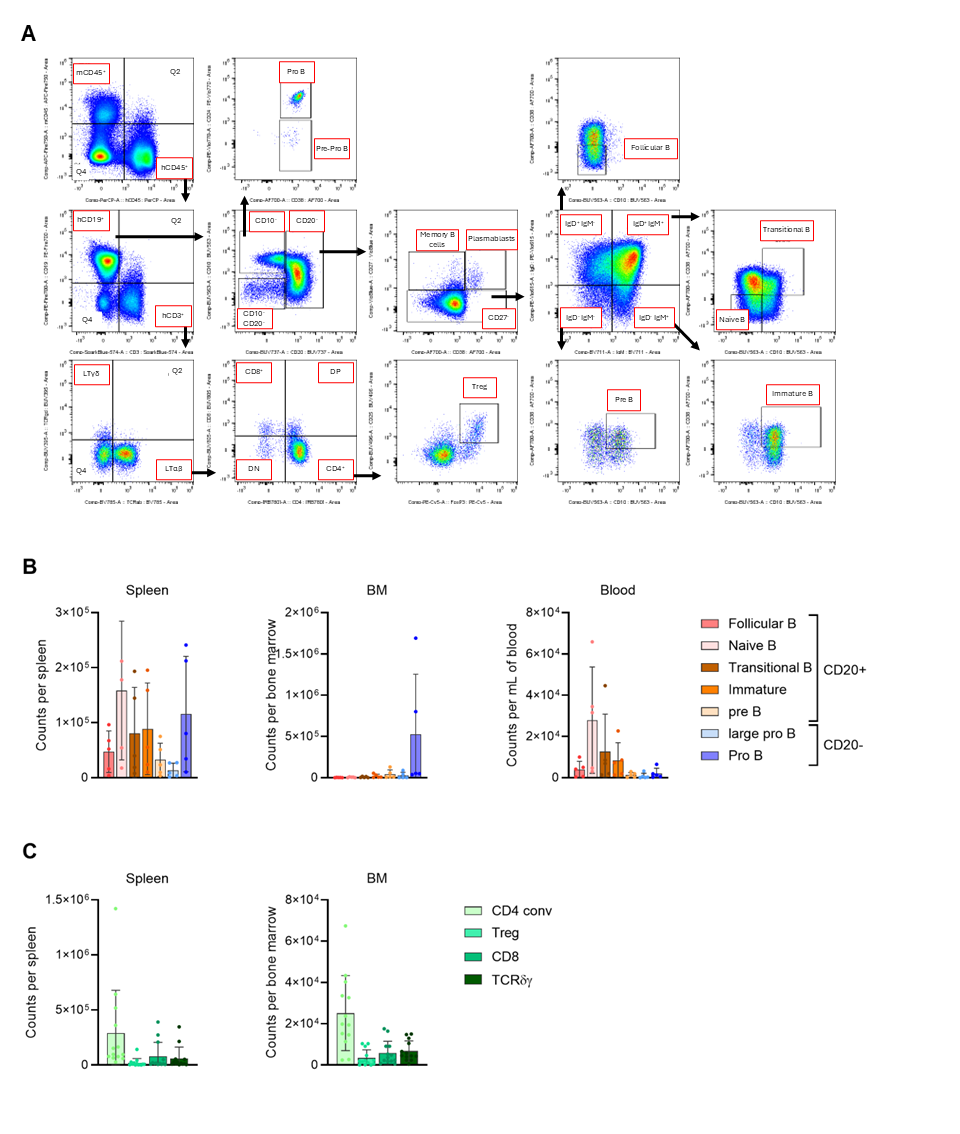


**Supplementary Figure 2 – Quantification of different subsets of lymphoid cells in genO-BRGSF-HIS mice. (A)** Gating strategy used to analyze the different cell populations.  **(B)** Total counts of the B cell subpopulations in the spleen, bone marrow (BM) and blood of hFlt3L-treated genO-BRGSF-HIS mice at 19 weeks of age (n= 5, 3 hCD34^+^ cell donors). **(C)** Total counts of the T cell subpopulations in the spleen or bone marrow (BM) of genO-BRGSF-HIS mice at 18 weeks of age (n=13, 6 hCD34^+^ cell donors), Number of cells per organ or per microliter of blood are represented. Error bars represent standard deviation. Each data point represents individual mice.

**Supplementary Figure 3 – genO-BRGSF-HIS mice develop different subsets of myeloid and dendritic cells upon hFlt3L treatment. (A)** and **(B)** Quantification of the different myeloid and dendritic cell subpopulations in the spleen of genO-BRGSF-HIS mice (n=9-11) at 16 weeks of age, following hFlt3L or vehicle (control) treatment, represented as a percentage relative to hCD45^+^ cells **(A)** or as total cell counts (cells/mL) **(B)**. Horizontal lines represent the mean. Open or filled squares represent 2 different hCD34^+^ cell donors, 5-6 mice per donor were analyzed. Data were analyzed using a Mann Whitney test. **P<0.01, ***P<0.001, ****P<0.0001, ns – non-significant.

**Supplementary Figure 4 – Human myeloid cells in the genO-BRGSF-HIS model express different PRRs.** Expression of TLR4 and TLR8 in different myeloid cells analyzed in the bone marrow (BM) and spleen, measured by cytometry at 36 weeks. genO-BRGSF-HIS mice (n=4) were reconstituted with hCD34^+^ cells from 3 different donors. Horizontal lines represent the mean.

**Supplementary Figure 5 – STING stimulation leads to T cell activation.** T cells (hCD3^+^) isolated from nine hFlt3L-treated genO-BRSGF-HIS mice were treated with 2’3’ cGAMP at 50 µg/mL, or left unstimulated (Unst). CD69 expression was measured 24 hours later by cytometry. Data were analyzed with a Welch T test. Bars represent standard deviation. 3 HSC donors were used, and 3 mice were used per donor. Individual data points represent pooled mice per donor. *P<0.05.

**Supplementary Figure 6 – MDA-MB-231 TME infiltration by human immune cells and its composition by hCD34^+^-donor. (A)** Quantification of the immune cell infiltration in the TME of MDA-MB-231 tumors per donor at 400-500 mm^3^. The percentage of murine or human CD45^+^ cells was calculated relative to total viable cells in the tumors. The graphs represent tumors in 5 hFlt3L-treated genO-BRGSF-HIS mice per donor, and the error bars represent standard deviation. **(B)** Cytometry quantification of the cell populations present in the human immune infiltrate of the MDA-MB-231 TME per donor, measured at two tumor volume timepoints. The graphs represent tumors in 2-3 hFlt3L-treated genO-BRGSF-HIS mice, and the error bars represent standard error of the mean.

**Supplementary Figure 7 – A549 TME immune cell infiltration and humanization rate vary according to tumor size. (A)** Quantification of the total immune cell infiltration in the TME of A549 tumors by tumor size. The percentage of murine or human CD45^+^ cells was calculated relative to total viable cells in the tumors. **(B)** Humanization rate in tumors represented by tumor size. Each point represents individual mice. Both graphs represent tumors in 6 genO-BRGSF-HIS mice per timepoint, implanted with 5 x 10^6^ A549 cells, and the error bars represent standard deviation.


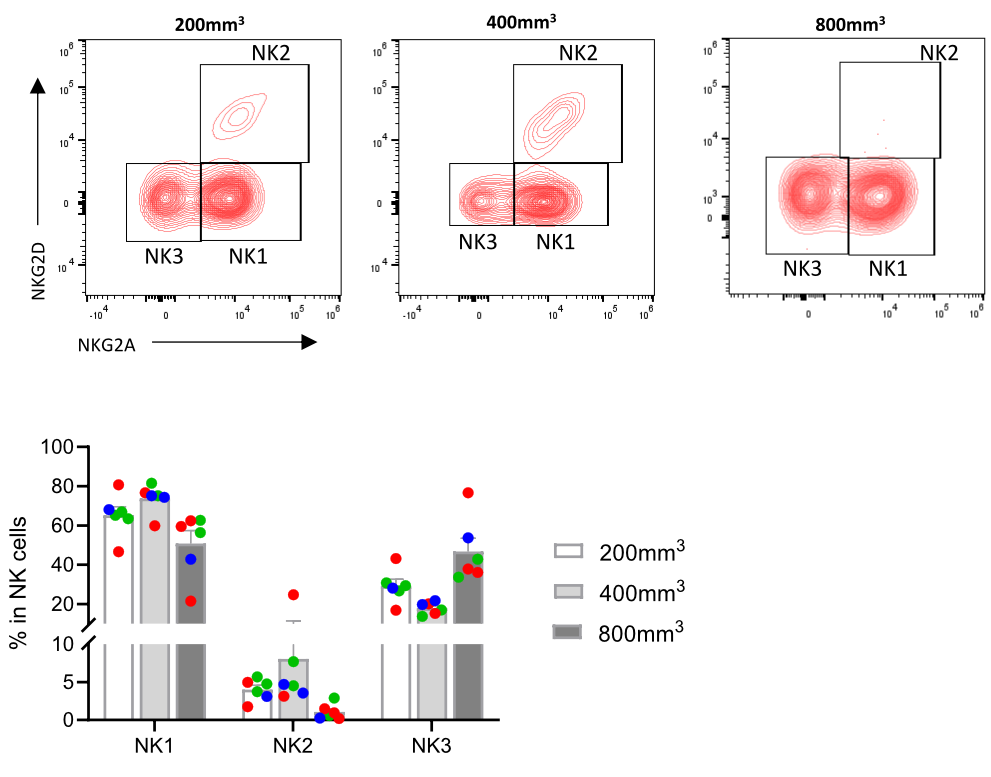


**Supplementary Figure 8 – The NK1, NK2 and NK3 subsets of NK cells are present in the A549 TME.** Representation of the gating strategy used to identify the different NK cell subsets and quantification of the percentages of NK subsets in the three timepoints. Graph represents tumors in 6 genO-BRGSF-HIS mice per timepoint, and the error bars represent standard deviation. Each point represents individual mice, and the colors represent different donors.

**Supplementary Figure 9 – The expression of NK cell markers in NK1, NK2 and NK3 subsets varies according to tumor size.** Quantification of the percentages of NK subsets expressing different NK cell markers, in three tumor-growth timepoints. Each graph represents tumors in 6 genO-BRGSF-HIS mice per timepoint, implanted with 5 x 10^6^ A549 cells, and the error bars represent standard deviation. Each point represents individual mice, and the colors represent different donors. nd – not detected. Data were analyzed with a two-way ANOVA. *P<0.05, **P<0.01, ***P<0.001.

**Supplementary Figure 10 – genO-BRGSF-HIS mice are permissive to engraftment with HPAF-II cells. (A)** Tumor growth was measured over time in genO-BRGSF-HIS mice engrafted with hCD34^+^ cells from 4 donors and implanted with 1.5 x 10^6^ HPAF-II cells. 5-6 mice were used per donor. **(B)** Mouse body weight was quantified following implantation with 1.5 x 10^6^ HPAF-II cells in genO-BRGSF-HIS mice. Mouse body weight is represented per hCD34^+^ HSC donor. 5-6 mice were used per donor. Error bars represent standard deviation.

**Supplementary Figure 11 – The HPAF-II TME infiltration by human immune cells and its composition are not hCD34^+^ HSC-donor dependent. (A)** Quantification of the immune cell infiltration in the TME of HPAF-II tumors per donor at 400-500 mm^3^. The percentage of murine or human CD45^+^ cells was calculated relative to the viable cells in the tumors. The graphs represent tumors in 3-6 genO-BRGSF-HIS mice implanted with 1.5 x 10^6^ HPAF-II cells. **(B)** Cytometry quantification of the cell populations present in the human immune infiltrate of the HPAF-II TME per donor. The graphs represent tumors in 3-6 genO-BRGSF-HIS mice. Error bars represent standard deviation. Each data point represents individual mice.
